# Advancements in Hydrazide-Based HDAC Inhibitors: A Review of Recent Developments and Therapeutic Potential

**DOI:** 10.1021/acs.jmedchem.5c01677

**Published:** 2025-07-10

**Authors:** Alessia Raucci, Clemens Zwergel, Sergio Valente, Antonello Mai

**Affiliations:** † Department of Drug Chemistry and Technologies, 9311 Sapienza University of Rome, Piazzale Aldo Moro 5, Rome 00185, Italy; ‡ Pasteur Institute, Cenci-Bolognetti Foundation, Sapienza University of Rome, Piazzale Aldo Moro 5, Rome 00185, Italy

## Abstract

Histone deacetylase (HDAC) impairment is strongly related to various cancers as well as neurodegenerative and inflammatory diseases. Considering HDACs’ crucial role as drug targets, over the past decade, the development of HDAC inhibitors (HDACi) has gained significant interest in the pharmaceutical field. To date, five HDAC inhibitors have been approved by the FDA, and many are currently in clinical trials. Despite the common use of hydroxamic acid and 2-aminoanilide groups as zinc-binding groups (ZBGs) in HDAC inhibitors, concerns about their instability, toxicity, and low bioavailability have led researchers to explore alternative ZBGs. Recently, the hydrazide group has emerged as a promising alternative, offering potentially safer properties and fewer off-target effects. This perspective will discuss recent advancements from a medicinal chemistry point of view related to the hydrazide group’s role in HDAC inhibitor development, highlighting their pharmaceutical properties, biological activities, and potential benefits in reducing side effects.

## Graphical abstract

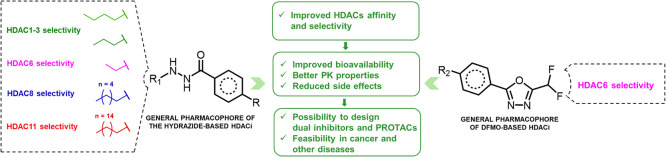

## Introduction

Histone deacetylases (HDACs) are crucial enzymes in regulating gene expression by removing acetyl groups from histone proteins. This deacetylation process influences the chromatin structure and, consequently, DNA accessibility for transcription. There are 18 HDAC isoforms, categorized into four main classes: I, II, III, and IV, each with specific biological functions and tissue distributions.^1^ HDAC dysregulation has been linked to various diseases, including numerous cancer types and neurodegenerative and inflammatory disorders.^1^ In cancer, HDAC function impairment can lead to the repression of tumor suppressor genes, promoting cell proliferation and survival.^2^ Considering the role of HDAC’s in cancer and diseases, growing interest has been observed over the past years in the pharmaceutical field regarding the discovery of HDAC inhibitors (HDACi).^3^ To date, several have entered clinical trials, while five of them have been approved by the FDA as drugs to be employed for cancer treatments (vorinostat, panobinostat, romidepsin, and belinostat) and for Duchenne therapy (givinostat). The pharmacophore model of HDACi is composed of a zinc-binding group (ZBG), a linker, and a cap group, which is validated by crystal structure^4^ and currently followed by researchers to develop new HDACi. The inhibiting properties in the HDAC inhibitors’ structure are exercised by the ZBG. ZBGs are implicated in chelating the zinc ion of the HDAC active pocket, thus causing its depletion and exerting the HDAC-inhibiting effect. HDACi can be classified according to their ZBG into hydroxamic acids, 2-aminoanilides, aliphatic acids, electrophilic ketones, and more.^5^ Among the developed HDACi, hydroxamic acid (HA) and 2-aminoanilide represent the most used ZBGs. HA is the first and most abundant ZBG used along the developed HDACi, related to its ability to impart a strong binding affinity for the catalytic zinc ion, resulting in a potent HDAC inhibitory activity.^6,7^ Indeed, four of the Food and Drug Administration (FDA)-approved HDACi bear the HA as ZBG (vorinostat, belinostat, panobinostat, and givinostat). In the past decade, researchers focused on optimizing the HDACi’s potency by developing derivatives currently under clinical investigation.^8^ Many derivatives have been developed by opportunely decorating the general pharmacophore of HA-based or 2-aminoanilide-based HDACi to achieve higher potency and higher selectivity toward the HDAC isoform of interest. Despite the potent efficacy displayed by the HA-based HDACi, many concerns have been raised by the scientific community about their clinical safety as extensive data from clinical trials conducted on the approved HDACi are now available.^9^ It has been shown that HA-based HDACi presented off-target effects due to their lack of selectivity toward HDAC isoforms and their affinity to many other metalloenzymes, such as iron–sulfur cluster proteins and the acyl-CoA hydrolase MBLAC2a, which have been reported as the most common collateral targets of the HA-based HDACi.^10^ Additionally, HA shows poor pharmacokinetic properties related to its rapid clearance. Indeed, hydrolysis metabolism reactions and conjugations conducted on HA, e.g., glucuronidation, lead to its depletion and rapid drug elimination. Glucuronidation also leads to the Lossen rearrangement, which is the main cause of the observed HA’s mutagenic properties. This process interconverts the hydroxamic group to an isocyanate that is susceptible to DNA residues’ attack, thus exerting potential genotoxicity and mutagenic activity.^11^

Another broadly used ZBG is the 2-aminoanilide group, which is contained in well-known clinically studied molecules such as entinostat and tucidinostat. Different from a great part of the HA-based HDACi, the 2-aminoanilide-based ones exhibit better selectivity among HDACs by preferring the inhibition of class I HDACs. In addition, a lower affinity toward other metal enzymes than the one proven by HA-based HDACi is held, thus reducing the 2-aminoanilide-based HDACi’s off-target effects.^12^ However, the presence of a free and exposed amino group within the 2-aminoanilide group represents a cause of potential toxicity *in vivo* for its susceptibility to become *p*-hydroxyaniline after undergoing *N*-hydroxylation and subsequent metabolic oxidation or rearrangement.^13^ Considering the highly electrophilic behavior of the former that can cause genotoxicity and hepatotoxicity by forming covalent adducts with DNA and proteins, which limits the clinical usage of 2-aminoanilides, no 2-aminoanilide-based HDACi has been yet approved by the FDA.^13^

Additionally, clinical data on the therapeutic use of HA-based HDACi have reported the occurrence of adverse cardiac side effects attributed to the inhibition of the hERG channel. This results in QT interval prolongation, which is a recognized risk factor for the development of fatal ventricular tachyarrhythmia episodes.^14^ Hepatic adverse effects have also been highlighted as significant concerns for this class of HDACi. Specifically, belinostat treatment has been associated with episodes of fatal hepatotoxicity. Further investigations into these adverse effects have highlighted the involvement of various HDAC isoforms in regulating the gene expression of key factors critical to cardiac and hepatic functions.^15^ The lack of isoform selectivity observed in FDA-approved HDACi, as well as in many HA-based inhibitors, is a primary contributor to these side effects. Developing isoform-selective HDAC inhibitors represents a promising strategy to mitigate these effects while enhancing therapeutic efficacy by targeting specific HDAC isoforms associated with distinct cancer types or other epigenetically driven diseases.

However, the above-discussed toxic profile related to HA and 2-aminoanilide, which can lead to undesirable effects,^16^ represents an important limit to overcome. Additionally, the PK analysis performed on HA-based HDACi reported low blood–brain barrier (BBB) permeability properties,^17^ thus limiting their applicability for central disease treatments. However, even though numerous studies have explored modifications in the linker and cap regions to improve the safety and selectivity of HA-based HDAC inhibitors,^8^ these changes might not fully mitigate the intrinsic limitations of HAs and 2-aminoanilides. This underscores the need for medicinal chemists to explore alternative ZBGs that offer improved safety profiles and enhanced potency, potentially overcoming drug resistance and improving patient outcomes. Consequently, over the past decade, the research on HDACi has increasingly shifted toward integrating alternative ZBGs. This approach offers a promising strategy to enhance pharmacokinetic properties and selectivity, potentially broadening the therapeutic applications of these molecules to target various cancer types and other diseases.^5^

## Alkylated Hydrazides

Among the novel ZBGs under investigation, the hydrazide moiety has emerged as a promising alternative to most ZBGs employed in HDACi development. This is supported by compounds already discussed in the perspective article published in August 2024,^18^ as well as the growing number of new compounds developed since then. The hydrazide group is present in several well-established drugs, such as isoniazid, an antituberculosis agent, and it is recognized for its therapeutic profile, including antioxidant,^19^ antimicrobial,^20^ and anti-inflammatory properties.^21^ Additionally, its safety profile has been extensively evaluated.^22^ The initial identification of a hydrazide-containing HDAC inhibitor was achieved through high-throughput screening (HTS) using the Scripps Drug Discovery Library by Wang et al., which isolated a 4-bromo-*N*’-butyl benzohydrazide, **UF010** (compound **1**), as a selective HDAC1–3 inhibitor.^23^ Considering the promising profile of the former, the Liao research group gave further insight into the kinetic and metabolic properties as well as the binding dynamics of the hydrazide-based HDACi by developing other derivatives.^5,24–27^ Notably, hydrazide-based HDACi exhibited slow-on/slow-off inhibition, forming stable complexes with HDAC enzymes, as evidenced by dilution experiments. Interestingly, the Michaelis–Menten kinetic studies performed on HDAC1 and HDAC3 by the Chou research group revealed that this new kind of ZBG confers mixed inhibition kinetics involving both competitive and noncompetitive sites. Indeed, additional computational docking studies performed on HDAC1 and HDAC3 reported that the binding is mediated through hydrogen bonding and hydrophobic interactions within both the active and allosteric sites, thus supporting the kinetics data about the mixed inhibition toward HDAC1 and HDAC3.^25,28,29^ Additionally, further assays regarding the pharmacokinetic profile of the hydrazide-based HDACi reported that it was negative in the Ames test for mutagenicity and demonstrated stability to glucuronidation in metabolism reactions,^22,26^ making it a safer alternative to HAs. Considering the emerging promising profile of hydrazide-based HDACi, other research groups focused on the development of these kinds of inhibitors. Notably, beyond its promising pharmacokinetic profile, the hydrazide ZBG has another fascinating property related to its ability to modulate HDAC isoform selectivity by simply changing the length of its alkylating chain. So, once achieved metabolic stability and selectivity among HDACs, the new hydrazide-based HDACi showed lesser off-target effects and a good selectivity among HDACs, thus offering a promising therapeutic potential in cancer and other disease treatments, even paving the way to extended clinical applications of HDAC inhibitors.^13,29^

It is well-documented that various diseases, including neurodegenerative and inflammatory disorders, are associated with impaired HDAC function.^1,30^ For instance, impairment of HDAC3 in Alzheimer’s disease exacerbates neurotoxic effects and disrupts cellular mechanisms.^31,32^ HDAC6 is implicated in oxidative processes that adversely affect neuronal survival.^33^ Furthermore, HDAC hampering has been linked to astrocyte- and T-cell-mediated inflammation in Parkinson’s disease.^34,35^ Indeed, considering the role played by HDAC inhibition in neurodegenerative diseases and the selectivity toward HDAC isoforms that could be achieved by different hydrazide alkylation grades, hydrazide-based HDACi are being explored for Alzheimer’s disease and Huntington’s disease treatments. Notably, the first biological evidence reports that modulation of gene expression and neuronal function were achieved.^1^ Moreover, hydrazide-based HDACi are being investigated in the treatment of inflammatory and fibrotic diseases due to the anti-inflammatory and antifibrotic effects via HDAC inhibition.^30^ Given the limitations of traditional HDACi with canonical ZBGs, such as HA and 2-aminoanilides, hydrazides may represent an intriguing approach for designing new HDACi with improved pharmacodynamic properties, including reduced side effects and enhanced BBB permeability and selectivity toward HDACs. This could facilitate the successful treatment of neurodegenerative diseases and other conditions by inhibiting fundamental epigenetic actors such as HDACs.

### Benzoyl Hydrazide-Based HDACi

Considering the promising role played by the hydrazide group in HDACi development, in this perspective, we will investigate the structure–activity relationship (SAR) properties of hydrazide-based HDACi by analyzing the pharmaceutical properties, biological activities, and the influence given by the chemically different types of hydrazide’s alkylation in conferring selectivity among HDACs, as reported in recent research papers (Figure 1).

**Figure 1:**
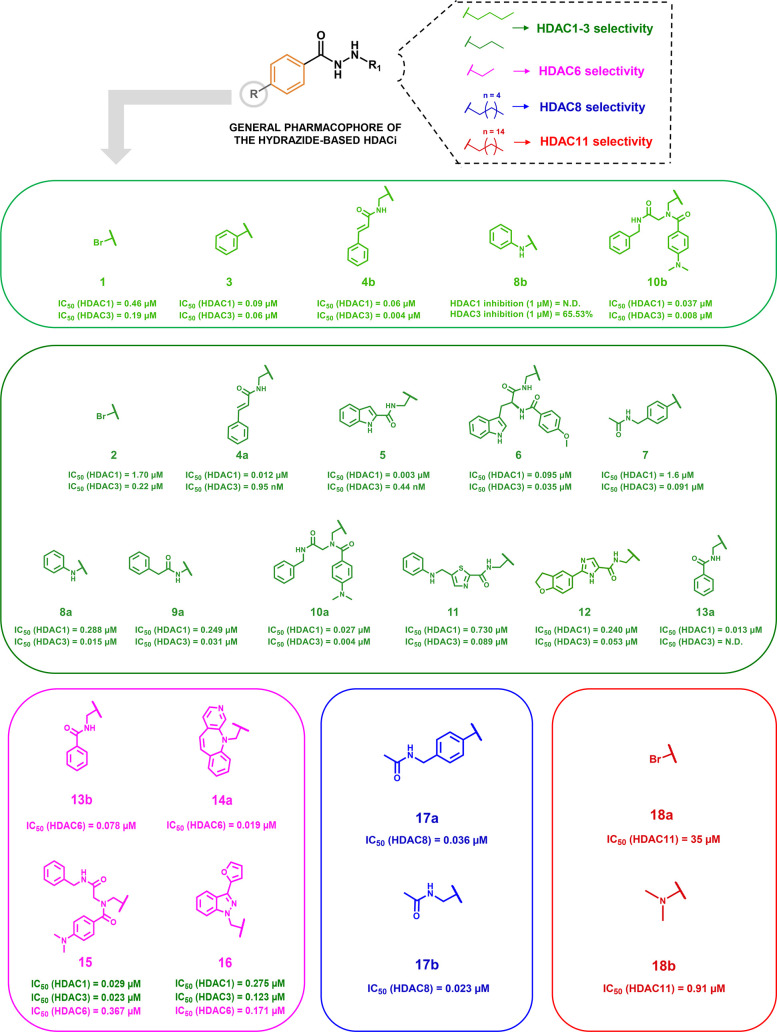
Chemical structures of hydrazide-based HDACi.

#### Class I HDAC-Selective Inhibitors

As previously discussed, the development of the hydrazide-based HDACi started in 2015 with the Liao research group, which identified hit compound **1** (**UF010**) from an HTS campaign. Compound **1** showed an HDAC1–3 inhibition in the low micromolar range (IC_50_ = 0.19–1.33 μM), in which a slight preference toward HDAC3 inhibition was observed (IC_50_ = 0.19 μM, selectivity index (SI) [HDAC1/HDAC3] = 2.4).^23^ Once the benzoyl hydrazide scaffold was individuated as the general HDAC-inhibiting pharmacophore, the same research group conducted further investigations regarding its SAR properties. Notably, it was noticed that elongation of the *N*-alkylating chain of the hydrazide moiety in compound **1** together with the introduction of aromatic rings led to a reduced HDAC-inhibiting potency and HDAC1–3 selectivity; however, the *N*-propyl hydrazide compound **2** (**SR3212**), an analog of compound **1**, exhibited similar HDAC3-inhibiting potency (IC_50_ = 0.22 μM). Compound **1**’s antiproliferative activity was observed in biological assays, and its selectivity toward HDAC1–3 was asserted by evaluating the acetylation rate obtained from Western blot assays in the HCT-116 cell line.

Additional docking studies reported a unique Zn^2+^ monodentate binding of the hydrazide ZBG of compound **1** while its butyl chain filled a deep hydrophobic foot pocket.

Once investigated the role played by the propyl/butyl hydrazide-alkylating chain in conferring class I HDAC selectivity, to explore the role given by the cap group in the benzoyl hydrazide scaffold, Liao’s research group further synthesized and evaluated several derivatives in which the bromine atom in compound **1** was replaced by several groups.^23^ In more detail, the introduction of electron-donating and/or electron-withdrawing groups (−OCH_3_, N­(CH_3_)_2_, −OCF_3_) did not affect the inhibiting profile of the hit compound **1**, while the introduction of other halogens (−F, −Cl) led to decreased potency and selectivity. The absence of substituents in the compound **1** *para*-position led to no inhibition up to 50 μM concentration. Notably, when a phenyl ring was inserted to obtain compound **3** (**SR3558**), enhanced HDAC1–3 inhibition potency (IC_50_ = 0.06–0.80 μM) was achieved, with preference toward HDAC1 and HDAC3 (IC_50_ = 0.09 μM and IC_50_ = 0.06 μM, respectively). Indeed, molecular docking studies performed on HDAC3 confirmed the former evidence regarding the better fit of the hydrazide’s butyl tail into the bottom space of the enzyme active site pocket, thus leading to a stronger inhibition by the above-mentioned substitutions. Interestingly, the previously discussed peculiar extra space differs among HDAC isoforms: it is absent in HDAC6, while it is narrower in other HDACs than in class I^27^ HDACs (Figure 2).

**Figure 2:**
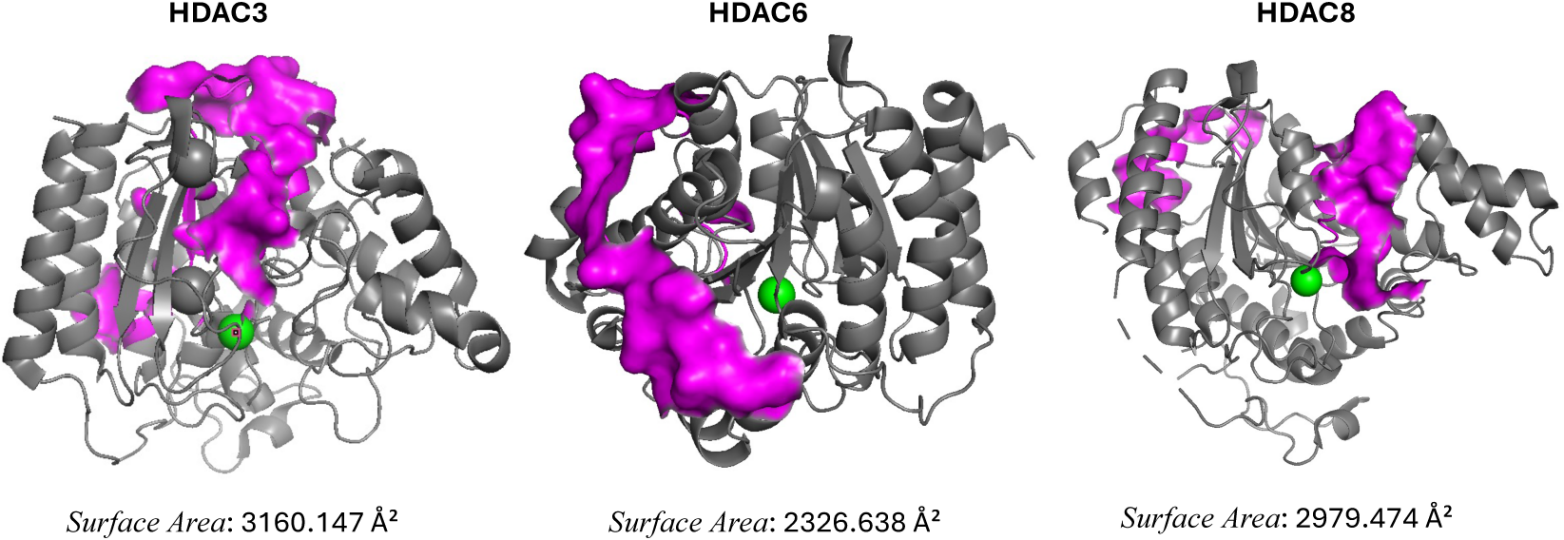
Crystal structures of HDAC3 (PDB ID: 4A69), HDAC6 (PDB ID: 5EDU), and HDAC8 (PDB ID: 1T69).

Since it was demonstrated that *N*-propyl and *N*-butyl hydrazide-alkylating chains could better fit into the extra pockets of these peculiar class I HDACs, the possibility of designing selective inhibitors for this HDAC class emerged. This insight led to the incorporation of such hydrazide alkylation patterns into new HDACi models.

In light of the first evidence about the role played by the hydrazide alkylation in determining HDAC selectivity, McClure et al. further shed light on the hydrazide SAR by exploring several hydrazide alkylations and different kinds of *p*-substituents on the benzoyl hydrazide pharmacophore model.^25^ Interestingly, it was noticed that among all the synthesized compounds, when an aliphatic cycle was inserted as a substituent on the hydrazide, less inhibiting properties and low selectivity were observed. In addition, the same trend was noticed when the hydrazide underwent a *bis*-alkylation. Notably, the previously discussed observations of Wang et al. were confirmed by the development of compounds **4a** and **4b**, which bear, respectively, an *N*-propyl- and *N*-butyl-hydrazide, and an *N*-methyl cinnamamide as the cap group. Indeed, compounds **4a** and **4b** exhibited class I HDAC selectivity, with a preference for HDAC3 inhibition (SI [HDAC1/HDAC3] = 12.4 and 16.4, respectively).^25^ Notably, higher HDAC3-inhibiting potency was achieved by compound **4a**, displaying an IC_50_ value in the subnanomolar range (IC_50_ = 0.95 nM), thus confirming the capability of the propyl chain to be better accommodated into the HDAC3 extra bottom space than its superior butyl homologous, and the influence given by the cap group’s decoration in impacting HDAC inhibition and selectivity. Although in the biological assays performed against HeK293 and HeLa cells, strong antiproliferative activity was displayed by compound **4a**, the presence of a Michael acceptor group in an α,β unsaturated ketone may induce related side effects for its high reactivity toward cysteines contained in various mammalian proteins. However, considering its role in determining potent HDAC3 inhibition, the same research group tried to optimize compound **4a** by stabilizing its Michael acceptor group. Indeed, Jiang et al., to abolish such a reactivity, attempted to cyclize the cinnamamide into a benzo-fused five- or six-membered ring, resulting in compound **5**, which bears a 1*H*-indole-2-carboxamide, as the best of their developed series.^29^ In line with its parent compound **4a**, compound **5** exhibited a selective inhibition profile toward class I HDACs (IC_50_ = 0.44 nM to 0.018 μM) while no HDAC4–11 inhibition was observed up to a 5 μM concentration. Compound **5** achieved around 2-fold higher potency against HDAC3 than its parent compound **4a** (IC_50_ = 0.44 nM vs IC_50_ = 0.95 nM, respectively) even though less selectivity toward HDAC3 was provided (SI [HDAC1/HDAC3] = 6.84). Further assays performed on Acute Myeloid Leukemia (AML) xenograft models showed a strong antiproliferative activity of compound **5** and to an improved pharmacokinetic (PK) profile (oral F% = 112%) than **4a** (oral F% = 19.8%).^29^

To deepen the understanding of the features given by various hydrazide alkylations and how selectivity and potency among HDACs could be achieved, the Chou research group further investigated these points by introducing alkylated hydrazides into their previously conceived 2-aminoanilide-based HDACi.^24,28^ Notably, the corresponding developed compounds provided higher class I HDAC selectivity than their 2-aminoanilide-based parent compound, especially when a propyl alkylating tail was inserted into the hydrazide group, thus reinforcing the former evidence of a better fit gained by the hydrazide *N*-propyl chain instead of the butyl one in the class I HDAC foot pocket. Indeed, compound **6** showed the best profile, being a class I HDAC-selective inhibitor with a 3-fold HDAC3 selectivity over HDAC1 (IC_50_ = 0.035 μM vs IC_50_ = 0.095 μM, respectively).^28^ Interestingly, further exploration of the inhibition mechanism of compound **6** and its 2-aminoanilide-based parent compound led to the identification of two different kinds of binding kinetic inhibition mechanisms against HDAC1–3. The former was a slow and mixed-type inhibitor toward HDAC1–3, while the latter displayed a competitive profile toward HDAC1–3. Notably, this evidence reinforced the increasing knowledge about the crucial role played by the alkylated hydrazide as ZBG is also influencing and changing the HDAC mechanism of inhibition with respect to the canonical and well-known above-discussed ZBGs.^28^ From the first knowledge obtained about the hydrazide-based HDACi, it was already clear that the insertion of a propyl or a butyl alkyl chain could confer class I HDAC-selective inhibition. However, the lack of selectivity among HDAC1–3 presented a limitation that must be overcome. Indeed, to achieve this goal and considering the pathological role played by HDAC3 in cancer and noncancerous diseases, researchers further investigated the influence of decorating the cap group of *N*-propyl/*N*-butyl hydrazide-based HDACi to obtain HDAC3-selective derivatives. Considering the reports about the 2-aminoanilide-based HDACi in which the presence of aromatic and aroyl groups as the cap functionality enhanced the HDAC1–3-inhibiting potency and selectivity,^36^ Sun et al. developed several derivatives of compound **3** in which chemically different substituents at the cap group were introduced.^37^ In particular, the best compound **7** from this SAR study, which bears a compound **3**-based *N*-methylacetamide-substituted structure and an *N*-propyl alkylation at the hydrazide group, displayed an improved HDAC3 inhibition selectivity over HDAC1 in comparison with its parent compound **3**, being 17-fold more selective over HDAC1 (IC_50_ = 0.091 μM vs IC_50_ = 1.6 μM). To better understand the influence of the cap group on achieving HDAC3-selective inhibition, further insights were obtained by performing docking studies of compound **7** in HDAC1–3 isoforms. It was proven that the peculiar class I HDAC foot pocket is narrower in HDAC3 than in HDAC1,2 due to the presence of the bulkier Tyr107 in HDAC3, thus explaining the more efficient insertion of the propyl chain in this area with respect to the butyl one. When an *N*-pentyl hydrazide-alkylated analog was developed, a drop of HDAC1–3-inhibiting potency was observed due to its difficult accommodation into the above-discussed foot pocket. Regarding the role played by the methylacetamide cap group of compound **7**, it was observed that it was solvent-exposed at the surface, but it made hydrogen bond interactions with the Asp93 of HDAC3.^37^

Pulya et al. focused as well on developing HDAC3-selective inhibitors by functionalizing the *para*-position of the benzoyl hydrazide by including different amino aromatic rings.^38^ Among their developed compounds, **8a**, bearing an *N*-propyl alkylated hydrazide and an aniline substituent at the *para*-position, resulted as the most potent HDAC3 inhibitor (IC_50_ = 0.015 μM) displaying a SI [HDAC1/HDAC3] of 18.7. The corresponding *N*-butyl-alkylated hydrazide analog **8b** exhibited lower selectivity against HDAC3 (% HDAC3 inhibition at 1 μM = 65.53% vs 97.48% of compound **8a**), thus confirming the previously discussed evidence regarding the *N*-propyl hydrazide role in influencing both potency and selectivity toward HDAC3.^24,25^ Interestingly, compound **9a**, which presents an anilido function at the *para-*position, exhibited good inhibiting properties against HDAC3 too (IC_50_ = 0.031 μM). To deepen the influence given by compounds **8a** and **9a**, cap group docking studies were performed on HDAC3, and a comparison with compound **1** interactions was conducted. Notably, it was noticed that the amine group of the aniline function of compound **8a** and the −NH of the amide group of compound **9a**, both directly bounded at the *para*-position of the general benzoyl-hydrazide pharmacophore, were involved in interactions with the negatively charged Asp93 of HDAC3 through a hydrogen bond, which is absent in compound **1** binding, thus resulting in a stronger binding with HDAC3 of both compounds **8a** and **9a**. Additionally, *in silico* studies revealed that compounds **8a** and **9a,** like compound **1**, exhibited a monodentate interaction within the catalytic Zn^2+^ ion, where the vicinal NH of the hydrazide was implicated in Zn^2+^ chelation and the distal NH interacts through a hydrogen bond with Tyr298. Indeed, in enzymatic assays, compound **8a** displayed more than 18-fold HDAC3 selectivity over other class I HDACs, while more than 3000-fold selectivity over HDAC4–6.^38^ In biological assays performed on the B16–F10 murine melanoma cell line and the 4T1 murine breast cancer cell line, compound **8a** showed high cytotoxic effects, whereas less cytotoxicity toward healthy human cell lines was observed. Additionally, compound **8a** profiled promising PK properties, and in *in vivo* assays, it exhibited promising antitumor activity in the 4T1-Luc xenograft model, which is strongly related to HDAC3 impairment, thus confirming compound **8a** as a promising candidate to be evaluated in such cancer types.^38^

Recently, further improvements of the compound **1** were achieved by the Hansen research group.^39^ Indeed, to optimize the PK and the inhibiting potency of their previously developed peptoid-based compound **VK1** ^**40**^, they replaced its 2-aminoanilide group with various alkylated hydrazides. Compounds **10a** and **10b**, bearing, respectively, an *N*-propyl- and an *N*-butyl-substituted hydrazide, resulted as the best compounds in class I HDAC-inhibiting terms, showing higher potency although low selectivity toward HDAC3 (IC_50_ = 0.004 μM, SI [HDAC1/HDAC3] = 6.75 and IC_50_ = 0.008 μM, SI [HDAC1/HDAC3] = 4.6, respectively). Considering the general SAR above-discussed in this perspective, a diminished inhibiting activity toward HDACs was observed when *bis*-alkylation or other heteroatoms were inserted among the hydrazide alkyl chain. Indeed, the Hansen research group presented a work in which their developed compounds differed only in the hydrazide’s kind of substitution and where it was confirmed that small and flexible types of hydrazide substitutions are more suitable for HDAC3 inhibition with respect to longer alkyl chains as well as with branched, rigid, and bulky substituents. Further docking studies reported that, differently from the previously discussed prototypes, compounds **10a** and **10b** interact within the HDAC3-Zn^2+^ in a bidentate manner, whereas both the distal N*H* and the carbonyl oxygen of the hydrazide are implicated in the chelation mechanism.^39^ The former evidence supports that monodentate or bidentate Zn^2+^ chelation could occur in HDAC inhibition by hydrazide-based HDACi, and the adoption of a kind of chelation is strongly influenced by the decoration of the whole molecule, which drives the positioning of the inhibitor inside the HDAC active pocket. The cytotoxicity of compounds **10a** and **10b** was then assessed in both native and cisplatin-resistant A2780 ovarian cancer and Cal27 head and neck carcinoma cell lines, and notably, the cisplatin resistance in these selected cell lines was completely overcome after treatment. Indeed, the treated cancer cell lines exhibited a major sensitivity to cisplatin administration, in which caspase 3/7-activation in apoptosis assays was detected in all the four cancer cell lines. Interestingly, to clarify the mechanism by which compounds **10a** and **10b** could increase the potency of cisplatin, assays regarding the detection of the increase of DNA damage (γH2AX formation) and the upregulation of p21 and Bcl-2-like protein 11 (BIM) upon the combination with cisplatin were performed in both Cal27/Cal27 CisR (cisplatin-resistant) cells. An increased level of γH2AX formation and p21 and BIM upregulation were observed upon combination treatment; hence, the major cisplatin sensitivity shown by the **10a**/**10b**-treated cell lines might be related to DNA damage. However, no DNA damage-related factors were induced in A2780/A2780 CisR (cisplatin-resistant) cells upon the combinations of HDACi **10a** and **10b** with cisplatin, concluding that other than DNA damage effects led to the apoptosis induction. Notably, when the combination was tested in a healthy cell line (HEK293), reduced cisplatin sensitivity was provided.^39^

The properties of the novel hydrazide-based HDACi were investigated with regard to PD-L1-overexpressing cancers. Indeed, Sun et al. developed compound **11 (HQ-30)**, an HDACi that bears an *N*-propyl hydrazide, which induced the degradation of PD-L1 by regulating cathepsin B (CTSB) in the lysosomes and by enhancing the infiltration of CD4+ and CD8+ T cells in the tumor microenvironment, thus activating antitumor immune activity.^41^ In particular, compound **11** showed better affinity for HDAC3 since being 8-fold more selective over HDAC1 (IC_50_ = 0.089 μM vs IC_50_ = 0.730 μM) and more than 100-fold over HDAC6–8. However, data regarding HDAC2 inhibition were missing. Docking analysis performed on HDAC3 reported a monodentate Zn^2+^ chelation, where the N*H* adjacent to the carbonyl group of the hydrazide chelates with the catalytic Zn^2+^ through a salt bridge, while the other amino group forms a hydrogen bond with the Gly143 residue.^41^ Considering the role played by HDAC3 in modulating PD-L1 expression in cancer,^42^ compound **11** was assayed on B16–F10 melanoma cancer cells, a PD-L1-overexpressing cancer cell line,^43^ and a reduced PD-L1 expression was highlighted, thus confirming the HDAC3-driven compound **11**’s mode of action. In addition, after compound **11** administration, T cell infiltration was also observed, thus suggesting that the former may exert its antitumor activity by activating the immune system. Considering the increasing interest in immunotherapeutics and the promising profile, such as the *in vivo* safety and drug-like properties held by hydrazide-based HDACi, this work may represent support regarding the possible clinical application of such compounds in immunotherapy, as well.

Sun et al. recently have reported the structure optimization of compound **11** to develop a more potent HDAC3 inhibitor with enhanced immunomodulatory activity.^44^ Molecular docking studies revealed that the cap group of **11** was solvent-exposed, making it amenable to structural modifications. A series of derivatives was synthesized, and compound **12**, featuring an imidazobenzofuran cap, exhibited the most promising profile. Compound **12** demonstrated a superior potency against HDAC3 (IC_50_ = 0.053 μM) compared to **11** (IC_50_ = 0.089 μM) and improved HDAC3 selectivity over HDAC1 (SI [HDAC1/HDAC3] = 75 vs 8 for HQ-30). Compound **12** was evaluated against multiple solid cancer cell lines, including B16–F10 (melanoma), HeLa (cervical carcinoma), and MC38 (colorectal carcinoma), and exhibited significant antiproliferative activity. Enzymatic assays confirmed the selectivity of compound **12** for HDAC3 over HDAC6, HDAC7, and HDAC8 (IC_50_ > 10 μM), although its inhibiting activity against HDAC2 remains uninvestigated. Western blot analyses performed on MC38 cells confirmed the former evidence by showing an upregulated level of Ac–H3 (substrate of HDAC3) and negligible effects on Ac-α-tubulin (HDAC6 substrate) or Ac-SMC3 (HDAC8 substrate) levels. *In vivo* studies using MC38 xenograft models demonstrated that SC26 effectively inhibited tumor growth. This effect was enhanced when compound **12** was combined with the PD-L1 inhibitor NP19.^45^ Further mechanistic studies revealed upregulation of PD-L1 expression in MC38 cells following compound **12** treatment, contrasting with the PD-L1 downregulation observed in B16–F10 cells treated with the parent compound **11**. These findings highlight the complex immunoregulatory effects of HDAC3 inhibition, which may be cancer cell type-dependent. Additionally, compound **12** exhibited favorable oral bioavailability and low toxicity, underscoring its potential as a promising epigenetic immunomodulator for cancer therapy.

#### HDAC6-Selective Inhibitors

As discussed, modifications at the hydrazide substitutions play a crucial role in defining selectivity profiles toward class I HDACs. Researchers have focused on developing inhibitors with specificity also for other isoforms. Specifically, based on the evidence that HDAC6, unlike HDAC1, lacks a large cavity beneath the zinc located in the enzyme’s active site (Figure 2), the potential to enhance HDAC6 selectivity was explored by incorporating smaller chains on the hydrazide-ZBG. Notably, the Chou group, starting from their derivative **13a**, which already exhibited class I HDAC inhibition but showed weak activity against HDAC6 (IC_50_ > 5 μM), developed a prototype with increased potency toward HDAC6 (IC_50_ = 0.078 μM) by shortening compound **13a**’s hydrazide alkyl chain of one carbon, thus yielding the corresponding ethyl-alkylated hydrazide compound **13b**.^46^ This finding prompted further modifications aimed to incorporate a large spatial cap group, acknowledging that the active site cleft linking the protein surface to the Zn^2+^ binding domain in HDAC6 is broader and shorter compared to other HDACs.^47^ This strategy led to the identification of compound **14a**, the most potent HDAC6 inhibitor in the series developed by the Chou research group. Compound **14a**, which features a tricyclic ring, displayed high selectivity for HDAC6 over class I HDACs (IC_50_ = 0.019 μM; SI [HDAC1/HDAC6] = 80). Interestingly, kinetic studies conducted on the former revealed distinct binding profiles for HDAC6 and class I HDACs. Specifically, compound **14a** exhibited a slow-on binding profile with HDAC6, whereas rapid-on properties were observed for HDAC1–3, likely due to the weak affinity of the ethyl hydrazide tail of compound **14a** toward the previously discussed HDAC1–3-foot pocket. Docking studies performed on HDAC6 reported a bidentate Zn^2+^ chelation of **14a**, where both the carbonyl group and N*H* of hydrazide were implicated within zinc interaction.^46^ Currently, many HDAC6 inhibitors face limitations regarding pharmacokinetic properties.^17^ Worthy of note, compound **14a** exhibited oral bioavailability of up to 93.4% and good BBB permeability, representing a promising alternative to address these limitations. Considering this evidence, along with HDAC6’s role in NLRP3 inflammasome activation,^48^ compound **14a** was evaluated in a lipopolysaccharide-induced endotoxic shock model in mice, in which NLRP3 plays a key role. Remarkably, oral administration of compound **14a** in the former model proved promising results, suggesting that compound **14a** could be a potential therapeutic agent for such conditions and may represent a novel HDAC6 inhibitor model with an improved pharmaceutical profile.

Even if the insertion of the ethyl alkyl chain on the hydrazide group could enhance the HDAC6 inhibition properties, a focus on the role played by the rest of the molecule must be considered too.

Indeed, the Hansen research group developed an HDAC inhibitor with potent anticancer properties bearing an ethylhydrazide ZBG which surprisingly turned out to be unselective. The compound was derived from a structure-based design study, starting from the peptide-based compound VK,^40^ in which variations in the linker region and types of ZBGs were investigated.^49^

Preliminary enzymatic inhibition assays conducted against HDAC1, HDAC3, and HDAC6 revealed that compound **15** (**DS-103**) exhibited the most potent inhibitory activity toward HDAC1 (IC_50_ = 0.029 μM), HDAC3 (IC_50_ = 0.023 μM), and HDAC6 (IC_50_ = 0.367 μM). Further inhibitory evaluations across a broader HDAC panel showed that the inhibition of HDAC1 and HDAC3 was 4–5-fold stronger than that of HDAC2 and approximately 13-fold higher than that of HDAC6. Weak inhibition was observed against HDAC8 (IC_50_ = 9.26 μM).

Interestingly, compound **15** is not a class I selective inhibitor, as selectivity is achieved when the hydrazide alkyl chain is elongated (e.g., butyl or propyl chain),^39^ as observed in their corresponding developed compounds **10a** and **10b** (above-discussed). This evidence confirms that the degree of alkylation of the hydrazide moiety plays a significant role in determining the selectivity profile of the corresponding HDAC inhibitor, although this is not a generally applicable rule. Indeed, in the case of compound **15,** other absent structural features are required to achieve HDAC6 selectivity such as the presence of a bulky cap group (like that of tubastatin A). This may explain the lack of HDAC6 selectivity of the former.

Compound **15** was selected for evaluation against Cal27 and A2780 cell lines, along with their respective cisplatin-resistant counterparts, Cal27 CisR and A2780 CisR, via MTT assays. Interestingly, a reversion of cisplatin resistance and increased sensitivity to cisplatin were observed in the resistant cell lines upon compound **15** treatment. The synergism between **15** and cisplatin was subsequently confirmed through analyses of caspase activation, apoptosis induction, and γH2AX expression. Additionally, compound **15** exhibited enhanced metabolic stability compared to its *N*-propyl analog (**10b**) and VK1. Furthermore, off-target effects were evaluated against other metalloenzymes (e.g., matrix metalloproteases and glutaminyl cyclase), and no significant inhibition was detected, confirming the HDAC selectivity of compound **15**. Western blot analysis revealed increased levels of acetylated histone H3 and acetylated α-tubulin, confirming the *in cellulo* target engagement of compound **15**.^49^

Interestingly, the Hansen group succeeded in obtaining the crystal structure of catalytic domain 2 of the HDAC6–compound **15** complex (PDB: 9DM6), representing the first-ever crystallographic structure of a hydrazide-based HDAC inhibitor bound to an HDAC enzyme (Figure 3). Structural analysis revealed that zinc chelation occurs through coordination of both the carbonyl oxygen and the ethylamino nitrogen of the ethylhydrazide moiety of compound **15**, forming a five-membered chelate ring that mimics the binding mode of hydroxamate-based inhibitors. The main difference between the two ZBGs lies in their charge: hydroxamates are negatively charged, whereas hydrazides are neutral. While involved in zinc coordination, the hydrazide ethylamino NH group and the carbonyl oxygen also engage in hydrogen-bonding interactions with H573 and Y745, respectively, thus reinforcing inhibitor binding within the HDAC active site.

**Figure 3:**
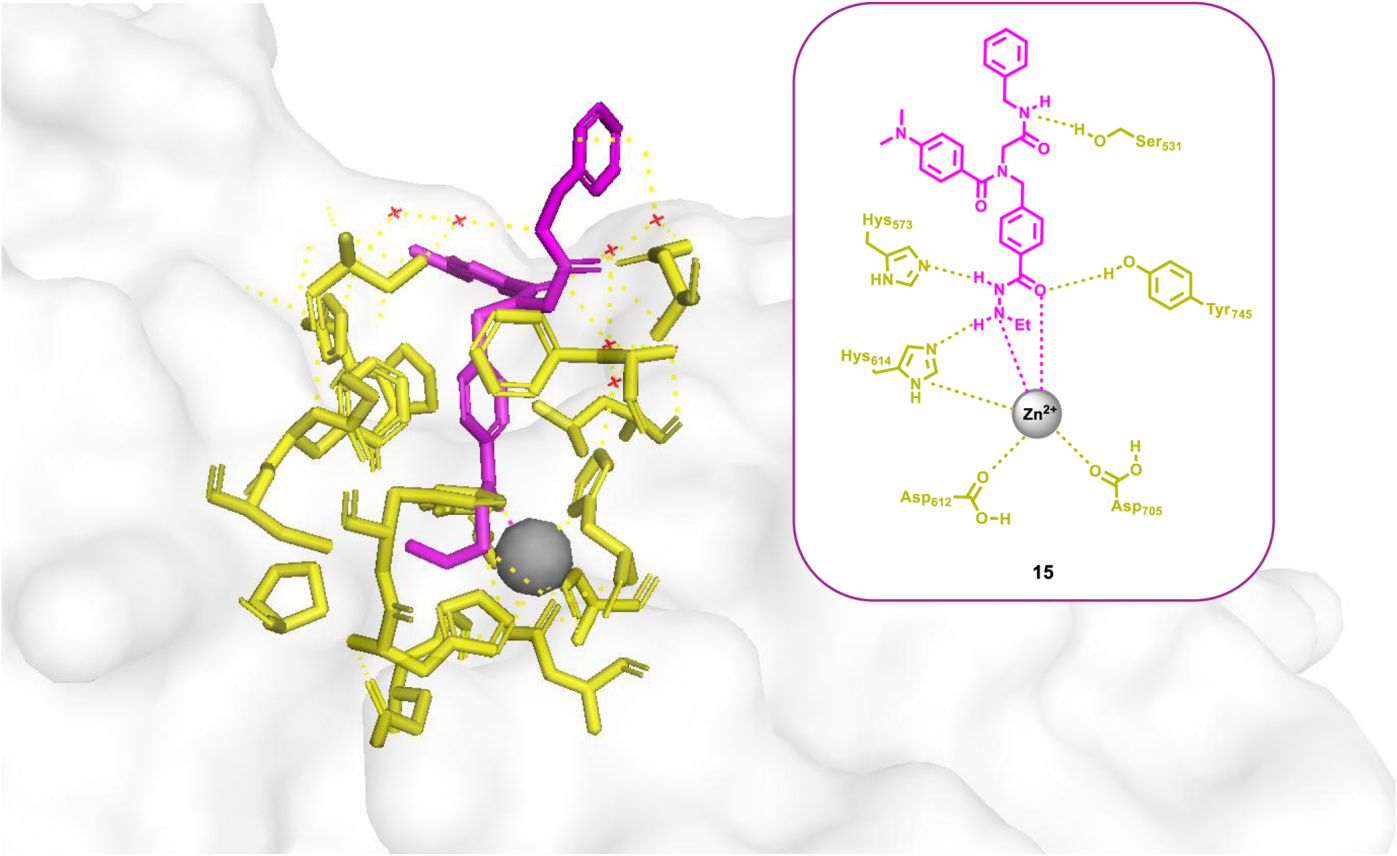
Crystal structure of the HDAC6–compound **15** complex (PDB: 9DM6).^49^

As discussed previously, the insertion of an *N*-alkyl hydrazide led to a better pharmacokinetic profile with respect to the corresponding hydroxamic acid–based analog. Indeed, Ali et al. developed compound **16**, an indazole-based HDACi bearing the compound **5j** structure (see ref. 50), which is the corresponding hydroxamic acid–based analog that showed a strong HDAC6-inhibiting profile (IC_50_ = 0.0029 μM), being over 25 times more selective over both HDAC1 and HDAC3 (IC_50_ [HDAC1] = 0.095 μM and IC_50_ [HDAC3] = 0.074 μM)^50^. Indeed, the researchers, in pursuit of improving the pharmacokinetic profile of the latter, inserted the *N*-ethyl hydrazide moiety, knowing that it may also lead to an improved HDAC6 inhibitory potency. The addition of this moiety was anticipated to augment the oral bioavailability of the compound, which indeed showed a notable enhancement (53% vs 1.2% of compound **5j**). Despite this improvement, a shift in the inhibitory profile was observed. Specifically, the potency against HDAC6 decreased (IC_50_ = 0.170 μM), and the selectivity toward HDAC6 was reduced, as evidenced by comparable inhibition of HDAC1 (IC_50_ = 0.275 μM) and HDAC3 (IC_50_ = 0.123 μM).^50^ This highlights the complex balance between pharmacokinetic improvements and potential compromises in selectivity and potency. While enhancing bioavailability is crucial, it is equally important to consider the impact of structural modifications on the overall activity profile.

#### HDAC8-Selective Inhibitors

It has been shown by Sun et al. that the elongation of the hydrazide alkyl chain length led to improved HDAC8-inhibiting properties, specifically when a hexyl tail is inserted on the hydrazide function. Indeed, the insertion of the former in their HDAC3-selective inhibitor **7** (above-discussed) resulted in compound **17a**, where a switched inhibitory profile, from an HDAC3-selective inhibition to an HDAC8-selective one, was observed (IC_50_ [HDAC8] = 0.036 μM and IC_50_ [HDAC3] > 20 μM).^37^ A SAR study was conducted, resulting in compound **17b** as a simplified version of **17a**, in which a benzene in the cap group was removed. The former exhibited a slightly improved inhibitory activity toward HDAC8 (IC_50_ = 0.023 μM), and it displayed a similar selectivity compared to compound **17a** toward the enzyme of interest. This additional evidence corroborates the role of the hydrazide alkylating chain length in determining a specific inhibition of an HDAC isoform through the interactions in the different-dimensioned foot pockets of HDACs. Once the high HDAC8 selectivity of compound **17a** was observed, it was assayed in T cells, considering the role played by HDAC8 in them.^51^ In more detail, hyperacetylation levels of two HDAC8 substrates, H3K27 and the structural maintenance of chromosomes 3 (SMC3) and upregulation of gene expression of several key memory- and effector-related transcription factors (TFs) and cytokines, were detected in CD4+ T lymphocytes, while CD8+ T cell proliferation and survival were observed. To assess the HDAC8 inhibition type of compound **17a**, enzymatic kinetic studies were performed, and, interestingly, a substrate-competitive behavior was unveiled.^37^

Next, docking poses of compound **17a** were performed within HDAC8 and HDAC3 to evaluate the crucial interactions leading to HDAC8 selectivity. In both selected HDAC isoforms, the hydrazide group of **17a** showed a bidentate chelation pattern with the catalytic zinc ion, while the biphenyl linker group was sandwiched between two pairs of phenylalanine residues (Phe144/152 and Phe200/208 in HDAC3/8, respectively). However, the hydrazide *N*-hexyl chain was not well accommodated within the HDAC3 foot pocket, because of clashing with the side chains of Met24, Arg28, and Leu133 in HDAC3, thus resulting in a strong decrease of HDAC3 inhibition. Interestingly, Leu133 and Met24 in HDAC3 were replaced by the bulky Trp141 and Ile34 in HDAC8, resulting in a larger foot pocket allowing the accommodation of the hydrazide *N*-hexyl chain of compound **17a**.^37^ This structural difference might explain the observed high HDAC8 inhibitory activity and selectivity of the former; however, these computational data need experimental confirmation, e.g., crystallography studies.

#### HDAC11-Selective Inhibitors

The exploration of the hydrazide-alkyl chain length has also been applied for targeting the HDAC11 isoform. Son et al., based on the observation that natural substrates of HDAC11 contain long fatty acid chains,^52^ identified the palmitic one, containing 16 carbon atoms, as an effective hydrazide-alkyl group to insert into the HDAC EIG to achieve selective HDAC11 inhibition.^53^ HDAC11 plays a crucial role in the regulation of serine hydroxymethyltransferase 2 (SHMT2), involved in Type I interferon signaling. Dysregulation of the former pathway is a crucial point in cancer,^54–56^ multiple sclerosis,^57^ viral infections,^58^ and metabolic diseases,^59,60^ thus underlining the high pharmaceutical interest to selectively target HDAC11. Son et al. first noticed that the insertion of an *N*-palmitoyl hydrazide into compound **1** yielded the corresponding hydrazide-based derivative **18a**, which showed a preference for HDAC11 inhibition.^53^ Indeed, the biochemical profiling of the former showed HDAC11 inhibition in the high micromolar range (IC_50_ = 35 μM), which was absent in the parent compound **1**. The introduction of the *N*-palmitoyl hydrazide into the vorinostat structure was also investigated, but no HDAC11 inhibition was detected. Consequently, to develop a stronger HDAC11 inhibitor, the research team conducted an optimization campaign by changing the 4-bromo benzene ring of compound **1** to other aromatic systems. Notably, the introduction of an electron-rich aromatic system, such as an *N,N*-dimethylaniline, led to the development of compound **18b**, displaying an improved HDAC11 inhibition (IC_50_ = 0.91 μM) comparable to that of a known HDAC11 inhibitor, FT895 (IC_50_ = 0.74 μM). Compound **18b**’s inhibition of HDAC1,4,8 was determined, and no activity was detected up to a concentration of 100 μM, thus exhibiting a higher HDAC11 selectivity than FT895. However, not all HDAC isoforms were assayed to verdict finally on the compound **18b** selectivity profile. Additional *in vitro* studies in MCF7 cells reported that compound **18b** was active in increasing the fatty acylation level of SHMT2, proving that the former was cell-permeable and could inhibit HDAC11 in a cellular context.^53^

### Hydrazides versus Known HA-Based HDACi

To elucidate the specific role of monoalkylated hydrazides in conferring inhibitory potency and selectivity across HDAC isoforms, researchers investigated this feature by introducing it into well-known HDAC inhibitors such as vorinostat and panobinostat^24^ (Figure 4). Li et al. reported a series of panobinostat-based derivatives incorporating various substituted hydrazides. Among them, compound **19**, which bears an *N*-propyl alkylation, exhibited selective inhibition within HDAC1–3, showing an HDAC3 selectivity index of 18-fold over HDAC1 (IC_50_ = 0.28 nM vs IC_50_ = 5.17 μM). Although compound **19** maintained significant inhibitory activity against HDAC1, the shift in selectivity spectrum compared to panobinostat underscores the crucial influence of the *N*-propyl hydrazide in promoting HDAC1–3 selectivity, a feature absent in the pan-HDAC inhibitory profile of panobinostat. Considering the antitumor activity of the latter in AML and the importance of class I HDAC inhibition in this cancer type, the antitumor efficacy of compound **19** was further investigated in MV4–11 cells. Compound **19** demonstrated an antiproliferative effect comparable to panobinostat, validating the capacity of the alkylated hydrazide to not only enhance HDAC selectivity but also confer potent cytotoxic activity against cancer cells.^24^

**Figure 4:**
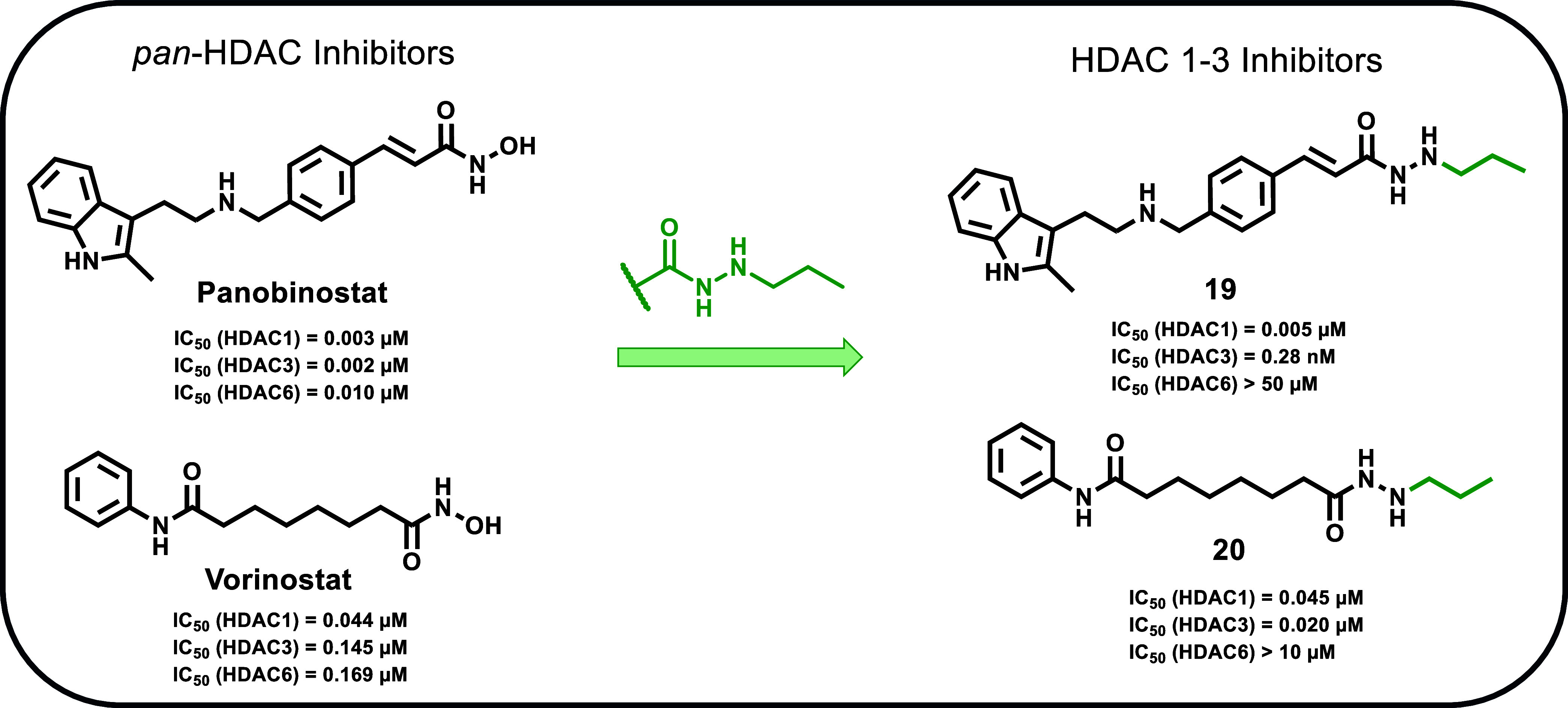
Chemical structures of panobinostat, vorinostat, and their corresponding hydrazide-based derivatives (compound **19** and **20**, respectively).

Yue et al. also investigated the role of monoalkylated hydrazide in the vorinostat structure. Of note, potency, selectivity, and enzymatic kinetics studies were evaluated, and key information about the influence given by the *N*-propyl alkylation was obtained.^27^ Compound **20**, the vorinostat *N*-propyl hydrazide-based analog, displayed selective inhibition toward HDAC1–3 in contrast to the pan-HDAC inhibition shown by vorinostat. Notably, in line with the general SAR data obtained from the *N*-propyl hydrazide-based HDACi already discussed above, compound **20** exhibited about 7-fold enhanced inhibition toward HDAC3 with respect to its parent compound vorinostat (IC_50_ = 0.020 μM vs IC_50_ = 0.145 μM, respectively). To further shed light on the role of *N*-propyl tail in this regard, additional kinetic studies on both compound **20** and vorinostat were performed, and as previously discussed in this perspective, compound **20** exhibited a slow-on/slow-off target interaction and a mixed inhibition behavior toward both HDAC1 and HDAC3, thus confirming the role played by the *N*-propyl hydrazide in conferring this kind of kinetic profile.^27^

### Variations at the Pharmacophore Benzene Ring

As extensively previously discussed, the benzoyl-hydrazide core, initially identified in compound **1**, served as the general pharmacophore for the development of the hydrazide-based HDACi. However, modifications of this general pharmacophore model were explored to further investigate its effects in HDACi-inhibiting terms (Figure 5). Pulya et al., revisiting their *N*-propyl hydrazide HDACi compound **8a** and their HDAC3 inhibitor prototype (**BG-45** ^61^), which bears a pyrazino-benzamide scaffold, developed a new series of hydrazide-based HDACi by merging these two moieties, thereby obtaining a pyrazole-hydrazide core instead of the benzoyl-hydrazide one.^62^ Notably, compound **21** emerged as the most potent prototype, exhibiting strong inhibitory activity and remarkable selectivity for HDAC3 (IC_50_ = 0.014 μM), being 141-fold more potent than against HDAC1 (IC_50_ = 1.983 μM) and 121-fold more potent than against HDAC2 (IC_50_ = 1.696 μM). Additionally, compound **21** displayed 700-fold selectivity over HDAC4–6 and HDAC8 isoforms. Hence, considering the pathological role of HDAC3 in triple-negative breast cancer (TNBC), the antitumor properties of compound **21** were investigated in this cell line, and a 40-fold selective antiproliferative effect toward breast cancer cells over healthy cell lines, such as human breast cells (MCF-10A), human embryonic kidney-293 (HEK-293), and human corneal epithelial cells (HCECs), was observed.^62^

**Figure 5:**
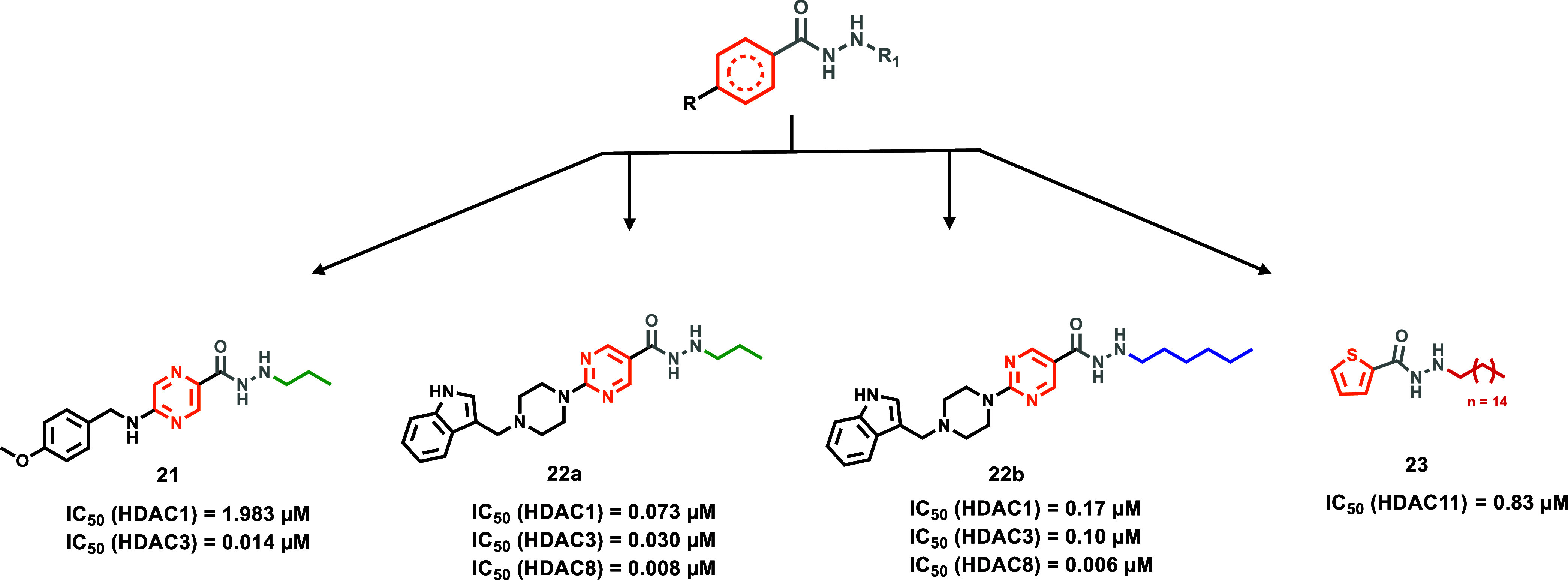
Chemical structures of compound **21**, **22a,b**, and **23**.

Along with these findings, compound **21** also exhibited a favorable pharmacokinetic profile, which supported its subsequent *in vivo* cytotoxic evaluation performed on 4T1-transfected mouse models. The promising biological profile regarding compound **21** was also given by the incorporation of a pyrazine ring, which played a crucial role in enhancing protein interactions.^63^ Indeed, further molecular docking studies were conducted, and it was observed that pyrazine was involved in the formation of three π–π stacking interactions with Phe144, Phe200, and His172 residues of HDAC3, thereby reinforcing the inhibitor’s interaction with the enzyme and facilitating the inhibiting monodentate interaction of the hydrazide’s vicinal N*H* with the catalytic Zn^2+^ ion through a salt bridge binding.

Other variations of the benzoyl-hydrazide core were investigated by Sun et al. As previously discussed, this research group developed compound **17a** as an HDAC8 inhibitor. However, this group also designed a series of derivatives in which the general benzoyl-hydrazide pharmacophore was replaced by a pyrimidinyl-hydrazide core.^37^ Specifically, they developed a pyrimidine-based inhibitor bearing an *N*-propyl hydrazide, compound **22a**, showing good inhibition of HDAC3 (IC_50_ = 0.030 μM) but lacked selectivity over HDAC1 and HDAC8 (IC_50_ = 0.073 μM and IC_50_ = 0.0082 μM, respectively). However, in compound **22b**, based on compound **22a** structure, where the *N*-propyl hydrazide group was replaced by an *N*-hexyl one, selectivity for HDAC8 over HDAC3 was achieved (IC_50_ = 0.0059 μM vs IC_50_ = 0.10 μM, respectively). Indeed, similarly to the work discussed above, docking studies reported that the incorporation of a nitrogen-based aromatic ring, such as the pyrimidine, plays a key role in enhancing interactions with HDAC8, thanks to its positioning into the hydrophobic tunnel, where it undergoes aromatic interactions. These kinds of interactions are also established in HDAC3: indeed, compound **22a** showed potent inhibitory activity against both HDAC8 and HDAC3, but HDAC8-selectivity was improved by introducing the *N*-hexyl alkyl chain at the hydrazide functionality in compound **22b**. Regarding the catalytic zinc ion chelation, both compounds interact in a bidentate manner, thus confirming the different kinds of chelation that can occur in function of the inhibitor disposition inside the HDAC active pocket.^37^

Son et al. developed together with compound **18a** another HDAC11 inhibitor by introducing an electron-rich aromatic system, such as a thiophene moiety, into the general hydrazide-based pharmacophore, thus obtaining compound **23** (**SIS17**), which however showed a very similar HDAC11 inhibition potency when compared to compound **18a** (IC_50_ = 0.83 μM and IC_50_ = 0.91 μM, respectively).^53^ On the other hand, compound **23** displayed high HDAC11-selectivity, indeed none of the other HDAC isoforms were inhibited up to 100 μM concentrations, whereas the reference HDAC11 inhibitor FT895 inhibited HDAC4 with a dual-digit micromolar level (IC_50_ = 25 μM) and HDAC8 with a single-digit micromolar level (IC_50_ = 9.2 μM). In further biological assays performed in MCF7 cells, compound **23** was more potent than compound **18a** and the reference FT895, due to its improved cell permeability and metabolic stability.^53^

Therefore, an additional consideration is that the general SAR for the benzoyl-hydrazide previously discussed is also maintained when the phenyl ring of the benzoyl-hydrazide pharmacophore is replaced by isostere heterocycles. These models have demonstrated significant potential in both *in vitro* assays and *in vivo* xenograft studies. Nonetheless, the characterization of these molecules has relied mainly on computational data, thus underscoring the need for experimental validation, e.g., crystallographic studies, to provide deeper insights into their structural and functional properties.

## Hydrazide-Based PROTACs

Proteolysis-targeting chimera (PROTAC) has emerged as a revolutionary technology in drug discovery^64^ thanks to its several advantages over conventional inhibitors, such as high potency, extended duration of action, and potential tissue/cell type selectivity, but importantly the capability to induce degradation of nondruggable proteins.^65^ In recent years, the interest toward PROTACs to apply on HDAC degradation has increased. Currently, a great part of the developed PROTACs targeting HDACs, despite being based on pan-HDACi, exhibit mainly a HDAC6-selective degradation.^66^ Indeed, the former is primarily located in the cytosol and can be targeted more easily than other HDACs found in the nucleus. This could at least partially explain the preference for HDAC6 degradation. However, this specificity limits the PROTAC approach, with HA able to degrade other HDAC isoforms. Considering the key role played by HDAC3 in cancer, the Liao research group investigated the possibility to achieve its selective degradation by developing an HDAC3-PROTAC (Figure 6).^67^ Specifically, they conjugated compound **3**,^23^ endowed with a certain HDAC3 selectivity, with the ligand for the Von Hippel–Lindau (VHL) E3 ubiquitin ligase at its terminal solvent-exposed phenyl ring, thus giving compound **24a** (**XZ9002**). This PROTAC potently and selectively degraded HDAC3 (DC_50_ = 0.042 μM, 14 h treatment) while sparing other HDAC isozymes in a triple-negative breast cancer cell line (MDA-MB-468) and a ER^+^ breast cancer cell line (T47D). Indeed, the performed Western blot assays reported that compound **24a** could induce HDAC3 degradation at a concentration of 0.1 μM in both assayed cancer cell lines, while negligible effects on the HDAC1 and HDAC2 levels were observed. The effects of compound **24a** on HDAC3 protein levels in MDA-MB-468 were long-lasting and reversible, thus determining the former as an inducer of HDAC3 degradation in a dose- and time-dependent manner.

**Figure 6:**
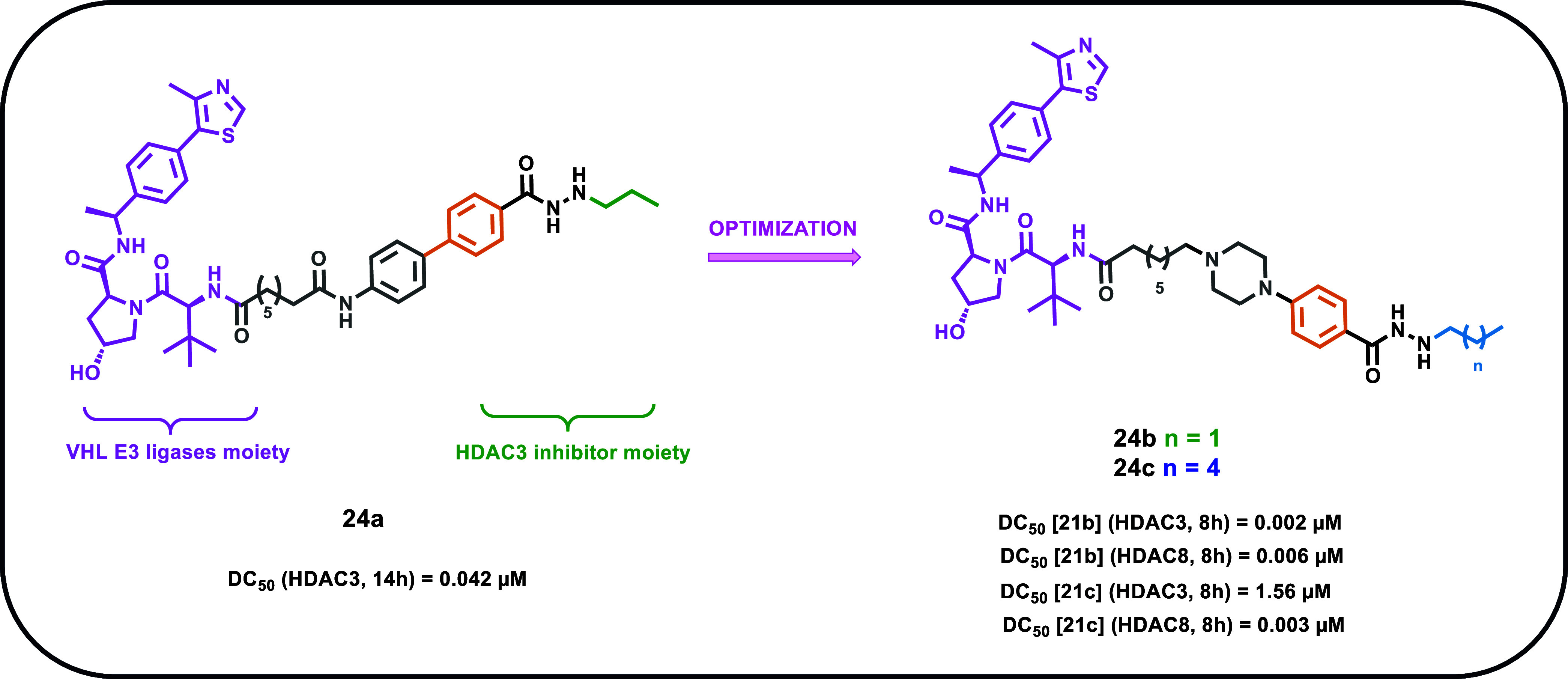
Chemical structures of compounds **24a**–**c**.

Then, compound **24a** was investigated regarding the survival and proliferation rates of breast cell lines using colony formation assays.^67^ Interestingly, compound **24a** was highly potent in suppressing the clonogenic growth of T47D, HCC1143, and BT549 breast cancer cells. Further enzymatic inhibition assays revealed that compound **24a** displayed moderate HDAC3 inhibition (IC_50_ = 0.35 μM), being less potent than compound **3** (IC_50_ = 0.06 μM). These findings confirm that the antiproliferative effects of compound **24a** were primarily associated with its HDAC3 degradation properties rather than HDAC3 inhibition.

Even though compound **24a** demonstrated a good HDAC3 degradation profile and promising activity against selected cancer cell lines, its *pan*-HDACi warhead represents a limitation that must be addressed. Specifically, the warhead was derived from compound **3**, which, due to its class I selective profile, was responsible for the class I inhibitory activity observed in compound **24a**. Considering that the DC_50_ of compound **24a** was 10-fold higher than its IC_50_ against HDAC3, and given its *pan*-class I HDAC inhibition, efforts were made to improve its physicochemical profile. The same research group performed a structural optimization study^68^ to develop a PROTAC able to selectively degrade HDAC3 at low concentrations while minimizing enzymatic inhibition of HDACs and off-target effects.

Notably, replacing the benzene group in the compound **3**-derived CAP moiety with a piperazine led to the development of compound **24b** (**YX968**), a dual HDAC3/8 PROTAC (Figure 6).^68^ Compound **24b** exhibited remarkable degradation efficiency, with DC_50_ values in the low nanomolar range for HDAC3 (0.0017 μM, 8 h) and HDAC8 (0.0061 μM, 8 h), while showing no degradation effect for HDAC1/2. Despite some inhibitory activity against HDAC1/2, the inability of compound **22b** to degrade these enzymes was likely due to the difficulty to form a productive ternary complex with HDAC1/2, compound **24b**, and VHL, as suggested by *in vitro* assays.

Interestingly, compound **24b** showed a ratio degradation DC_50_/HDAC inhibitory IC_50_ values for HDAC3/8 of 100, thus achieving a tool compound useful to explore the biological effects of selective chemical knockdown of HDAC3/8 without interference from *pan*-class I HDAC inhibition. Furthermore, compound **24b** provided significant antiproliferative activity in MCF7 and T47D cell lines, as well as in TNBC cell lines BT549, MDA-MB-468, and HCC1806.^68^

Recently, the same research group conducted a study aimed at identifying an HDAC8-selective PROTAC for further investigating its unique biological functions and therapeutic potential. Building on the observation that the foot pocket in HDAC8 is larger than that one in HDAC3, a key feature that enables selective inhibition of HDAC8 in hydrazide-based HDACi, they replaced the *N*-propyl chain of compound **24b** with an *N*-hexyl group (Figure 6). This modification led to the development of compound **24c (YX862)**, their most effective HDAC8-selective degrader.^69^ Compound **24c** achieved highly selective HDAC8 degradation at single-digit nanomolar concentrations (DC_50_ = 0.0026 μM), with 400-fold selectivity over HDAC3 degradation (DC_50_ = 1.56 μM). A ternary complex formation assay confirmed that compound **24c** gave robust results with HDAC8 but not with HDAC3. Moreover, compound **24c** displayed an HDAC8 degradation potency 75-fold more potent than its HDAC8 inhibitory activity (IC_50_ = 0.195 μM). Functionally, compound **24c** was demonstrated to possess strong antiproliferative effects in breast cancer cell lines (MCF7 and MDA-MB-231) and TNBC lines (BT549, MDA-MB-468, and HCC1806). Comparative studies using compound **24c**, the HDAC3/8 dual degrader compound **24b**, and the HDAC3-selective degrader compound **24a** revealed that combined HDAC8 and HDAC3 degradation synergistically enhances breast cancer cell death, whereas HDAC8 degradation alone does not affect their proliferation.^69^ Interestingly, HDAC8 degradation alone effectively suppressed the growth of diffuse large B-cell lymphoma (DLBCL) cells, with significantly greater potency than the widely used HDAC8 inhibitor PCI-34051.

These findings underscore the potential of HDAC8 degradation in the treatment of DLBCL and highlight its role in uncovering new biological and therapeutic insights related to HDAC8. However, the promising profile observed by this PROTAC series requires further experimental validation, such as *in* *vivo* studies, to gain deeper insight into its anticancer effects within more complex systems.

Recently, further improvements regarding the development of HDAC8-PROTACs were obtained by the Dekker research group. In particular, the authors developed a series of PROTACs able to selectively induce HDAC8 degradation^70^ (Figure 7). Among their developed compounds, **25 (Z16)** exhibited the highest level of HDAC8 degradation. Compound **25** embodies both the structure of the previously discussed HDAC8-selective inhibitor **17a** ^37^ and the *N*-aryl glutarimide, as the E3 ligase recruiter moiety, linked by a piperidine. Compound **25** showed the highest degradation rate regarding HDAC8 in T cell leukemia Jurkat cells, exhibiting a decrease of HDAC8 levels by 92% after 6 h of treatment with 0.1 μM of compound **25**. Notably, no HDAC1–4, HDAC6–7, and HDAC11 degradation was observed. This evidence underlined the good selectivity of compound **25** toward HDAC8 degradation among HDACs. In addition, the HDAC8-selective degradation induced by compound **25** was also observed in HCT-116, THP-1, and A549 cell lines by performing Western blot assays to evaluate the acetylation levels of SMC3, an HDAC8 intracellular target. Enzymatic assays showed that compound **17b** inhibited HDAC8 more effectively than compound **25** (IC_50_ = 0.036 μM vs IC_50_ = 2.7 μM). Since **25** is a PROTAC designed to degrade HDAC8 rather than inhibit it, its antiproliferative effect is likely due to HDAC8 degradation rather than direct inhibition.

**Figure 7:**
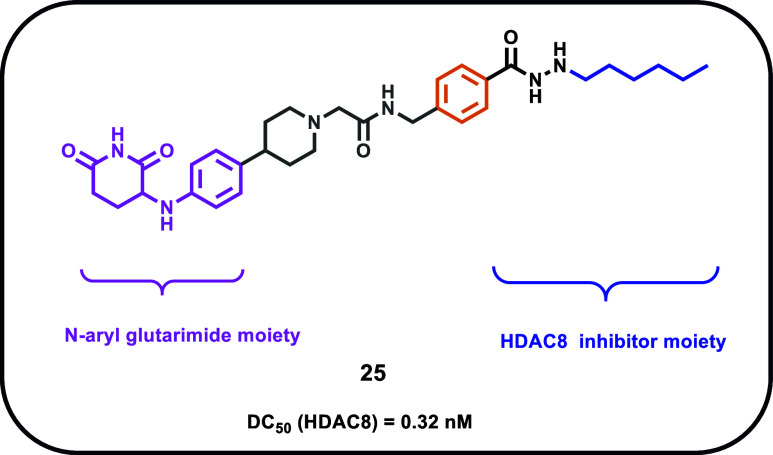
Chemical structure of compound **25**.

Recently, the Hansen research group have reported the development of a series of HDAC6-selective PROTACs, in which an ethyl hydrazide moiety was strategically inserted to enhance selectivity toward HDAC6 degradation.^71^ To develop their compounds, the researchers merged the HDAC-interacting pharmacophore derived from compound **A6** (see ref. 72) with two different E3 ligase recruiters: 6-fluorothalidomide and VH032-amine, using a variety of linker types. First, *in vitro* inhibition assays were conducted to evaluate the activity of the compounds against HDAC1–3 and HDAC6. Generally, all compounds exhibited no selective inhibition for the HDAC isoforms tested, but a certain cytotoxicity was observed in the multiple myeloma cell line MM.1S. Notably, the thalidomide-based PROTACs demonstrated moderate cytotoxicity, with compound **26** emerging as the most promising candidate within the series (Figure 8). Interestingly, and different from the biochemical inhibition profile, compound **26** selectively induced HDAC6 degradation, which was further confirmed via immunoblot analysis. In detail, **26** exhibited the highest degradation efficiency, with a *D*_max_ of 91% at 0.5 μM and a DC_50_ of 0.014 μM. Additional immunoblot experiments compared **26** with its thalidomide-methylated, nondegrading analog NC-**26** (see ref. 71), which lacks the structural features required for CRBN recruitment and subsequent HDAC6 degradation. Notably, hyperacetylated α-tubulin was detected only in cells treated with **26**, thereby confirming the necessity of a free imide group for effective CRBN engagement and HDAC6 degradation.^71^ Additional chemoproteomic analyses conducted in MM.1S cells revealed that other than HDAC6, compound **26** induced degradation of MIER1, a component of the HDAC1/2 complex. However, HDAC1/2 was not degraded, supporting the conclusion that **26** remains a selective HDAC6 degrader. To further investigate why the VH032-based PROTACs (see ref. 71) failed to induce HDAC6 degradation despite their inhibitory activity in enzymatic assays, molecular docking studies were performed to compare the formation of ternary complexes involving HDAC6, CRBN, and the PROTACs. Specifically, the binding mode of **26** exhibited a higher docking score, suggesting that the insufficient ternary complex formation observed with the VH032-based compounds is the likely cause of their reduced degradation efficacy.

**Figure 8:**
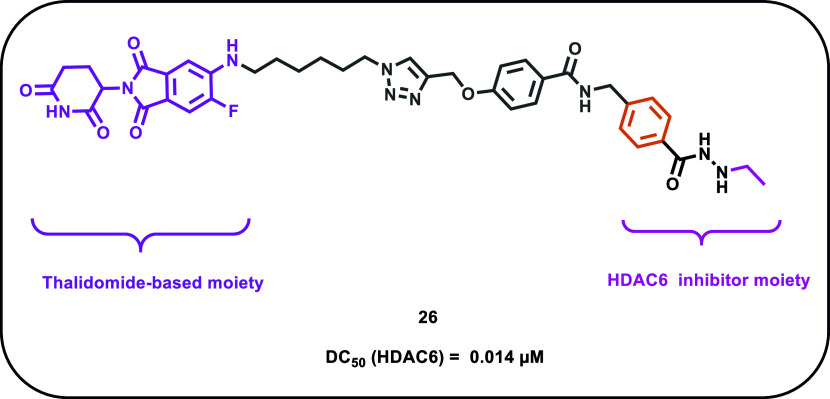
Chemical structure of compound **26**.

## Hydrazide-Based Dual Inhibitors

The development of dual inhibitors represents a significant advancement in research for their enhanced therapeutic efficacy and potential synergistic effects that could provide.^73^ Considering the good pharmacokinetic and pharmacodynamic profile given by the monoalkylated hydrazide among the HDAC inhibitors, its role was also investigated in the dual inhibitor development (Figure 9). Xue et al. first focused on this issue by developing an HDAC/Vascular Endothelial Growth Factor (VEGFR) dual inhibitor prototype by merging an *N*-propyl-benzoyl hydrazide with a pazopanib-based moiety as the VEGFR-inhibiting pharmacophore.^74^ Compound **27** (Figure 9) exhibited the most promising profile, displaying HDAC1 selectivity (IC_50_ = 0.42 μM) over HDAC4, HDAC6, and HDAC11 (IC_50_ = 8.96 μM, IC_50_ > 10 μM, and IC_50_ > 10 μM, respectively) and a favorable VEGFR inhibition profile (IC_50_ = 0.025–0.037 μM). Although inhibition data against HDAC2 and HDAC3 were unavailable, the SAR properties of the monoalkylated hydrazide scaffold appeared to be preserved within this dual-targeting molecule. Further biological experiments in various cancer cell lines (e.g., HCT-116, MDA-MB-231) highlighted the antiproliferative potential of compound **27**. However, additional structural optimization would be necessary, as the compound did not demonstrate superior metabolic stability compared to that of pazopanib. Nevertheless, these findings establish hydrazides as a promising scaffold for improving pharmacological properties in dual-target inhibitors, a hot topic in current medicinal chemistry research.

**Figure 9:**
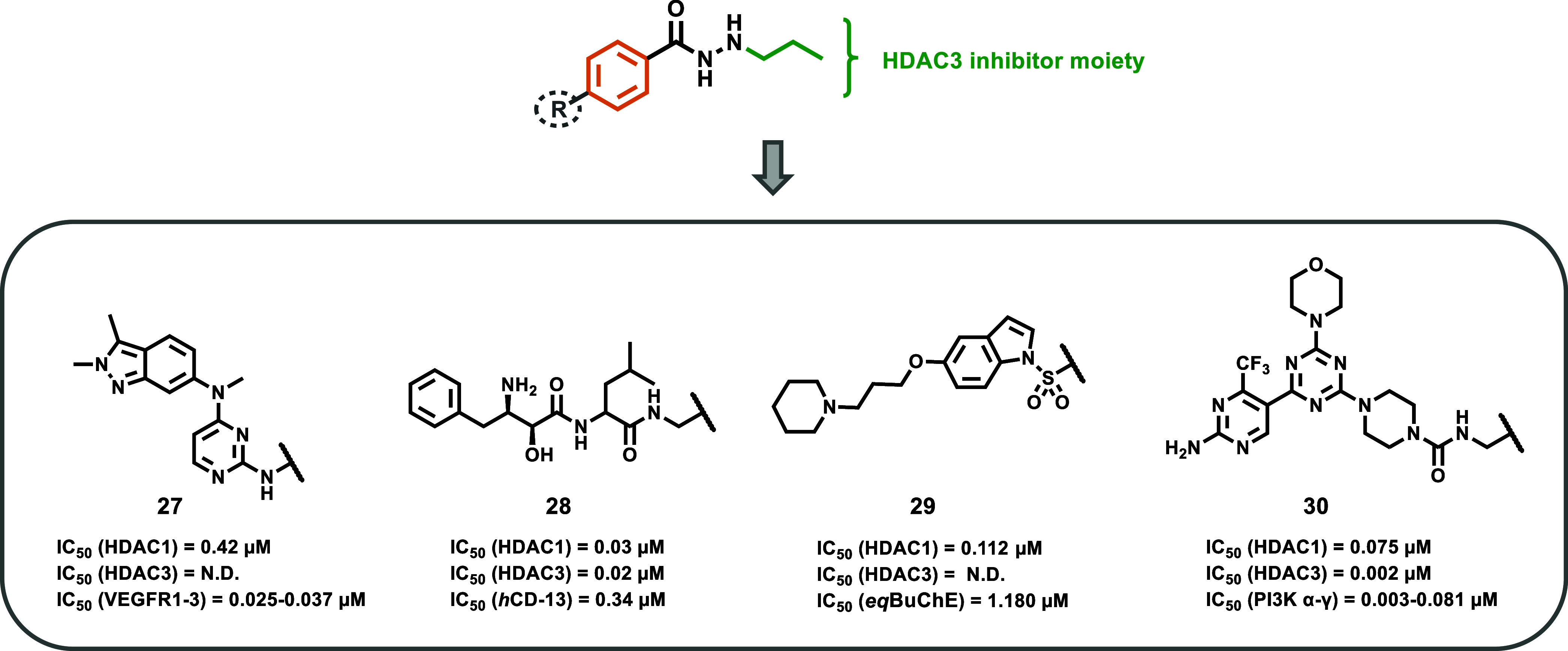
Chemical structures of dual inhibitors **27**–**30**.

CD13 (or aminopeptidase N, APN) is a transmembrane glycoprotein whose elevated expression in tumors is related to a major invasive and metastatic behavior.^75^ The combination that could be given by the CD13 inhibition with the antiproliferative and cytotoxic effects of HDAC inhibition could result in improved efficacy in cancer treatment.^76^ Jia et al. explored the incorporation of *N*-monoalkylated hydrazides within HDAC-based dual inhibitors, developing compound **28** as their most promising HDAC/CD13 dual inhibitor.^77^ Compound **28** proved to have strong antiproliferative activity against MV4–11 and A549 cell lines, outperforming the reference compound vorinostat. However, a direct comparison with vorinostat/betastin (a CD13 inhibitor) combination was not made; thus, the study is lacking an important evaluation for assessing the potential superior effect of compound **28**. Consistent with the SAR trends above-discussed in this perspective, compound **28** exhibited a class I selectivity profile (HDAC1–3; IC_50_ = 0.02–0.06 μM) with more than 100-fold selectivity over HDAC8 (IC_50_ = 4.65 μM) and HDAC11 (IC_50_ = 1.24 μM). The inhibition of HDAC1–3 was attributed exclusively to the hydrazide, since a non-HDAC inhibitor analog of compound **28**, bearing a methyl ester instead of the hydrazide moiety, showed no HDAC inhibition. Mechanistic cytotoxicity studies indicated that compound **28** was able to induce cell cycle arrest at the sub-G1 phase in MV4–11 cells, while it was able to induce cell cycle arrest at the G2/M phase in A549 cells. Importantly, only minimal cytotoxicity against healthy liver cells (HL7702) was observed at over 100 μM concentrations, underscoring cancer cells’ selectivity feature. *In vitro* stability assessments revealed that compound **28** was stable in human plasma, artificial gastric, and intestinal juices.^77^ This stability further supports the advantageous properties of the alkylated hydrazide, validating its improved pharmacokinetic properties with respect to HA as HDAC inhibitors.

Recently, the hydrazide moiety has also been incorporated into hybrid molecules for targeting neurodegenerative diseases. Toledano et al. explored the HDAC-inhibiting potential of an *N*-propyl hydrazide within a multitargeting small molecule derived from the merging of contilisant and tubastatin A, replacing the HA of the latter with a propyl-alkylated hydrazide.^78^ Consistent with the SAR trends of the latter, the insertion of a propyl chain in the hybrid compound **29** resulted in a multitargeting derivative that exhibited potent HDAC1 inhibitory activity at the nanomolar level (IC_50_ = 0.112 μM), showing more than 90-fold selectivity over HDAC6. However, further inhibition data against HDAC2, HDAC3, and other isoforms remain unavailable. Although the research group prioritized other hybrids with HDAC6-targeting profiles and different ZBGs (see ref. 78) for biological evaluation, the findings reinforce the previous hydrazide SAR that is maintained even in dual/multitargeting inhibitor designs. Nevertheless, a deeper investigation into the chemical and biological profile of compound **29** would be required to fully characterize the therapeutic potential in neurodegenerative diseases of such hydrazide-based dual inhibitors.

Among recently reported HDAC-based dual inhibitors, Hou et al. described a promising morpholino-triazine scaffold-based dual PI3K/HDAC inhibitor, compound **30**, which emerged as the most potent molecule within their series.^79^ Given the well-established interplay between PI3K and HDAC signaling pathways in cancer and the encouraging preclinical results of fimepinostat, a hydroxamic acid-based PI3K/HDAC dual inhibitor,^80^ the authors rationally designed their compounds by integrating an *N*-propyl hydrazide moiety into the PI3K-targeting pharmacophore derived from ZSTK474, a *pan*-Class I PI3K inhibitor, which is currently in clinical phases I and II,^81^ by replacing the solvent-exposed morpholine ring of the latter. Compound **30** displayed potent inhibition properties against both targets. In particular, it demonstrated broad inhibitory activity across PI3Kα–γ isoforms (IC_50_ values ranging from 0.0025 to 0.0805 μM) and notable selectivity for class I HDACs (HDAC1 [IC_50_ = 0.0755 μM], HDAC2 [IC_50_ = 0.0709 μM], and especially HDAC3 [IC_50_ = 0.0019 μM]), while showing minimal activity against HDAC4–7, 9, and 11 (IC_50_ > 10 μM). In cellular assays, **30** exhibited significant antiproliferative activity in various cancer cell lines including MV4–11, Jeko-1, HL60, and MCF-7. Notably, in MV4–11 cells, compound **30** outperformed both vorinostat and ZSTK474 used as monotherapies, indicating a potential synergistic effect resulting from its dual-targeting capability. Apoptosis induction studies in Jeko-1 cells further supported its efficacy, with **30** showing stronger pro-apoptotic effects compared to the combination of ZSTK474 and compound **39a** (see ref. 79). The latter, a derivative of **30** in which the morpholine ring, critical for PI3K binding, was replaced with a piperidine one, lacks PI3K inhibitory activity and served as a positive control of HDAC inhibition. Despite these encouraging results, compound **30** showed overall lower antiproliferative activity compared to fimepinostat. This discrepancy may stem from differing physicochemical properties, particularly those affecting membrane permeability and intracellular accumulation. Consequently, further investigation of the pharmacokinetic profile and *in vivo* efficacy studies in murine models are warranted to better assess the therapeutic potential of compound **30** as a novel PI3K/HDAC dual inhibitor.

Recently, the Guo research group successfully implemented the strategy of introducing an *N*-alkylated hydrazide as a ZBG to develop novel dual PD-L1/HDAC3 inhibitors. In particular, the research team, starting from the binding mode analysis of Wang-24 (a PD-1/PD-L1 disruptor, see ref. 82) and compound **4a** with PD-L1 and HDAC3, respectively, identified that the surface recognition cap (SRC) of compound **4a** could be replaced by the pyrido-biphenyl structure of Wang-24.^83^ This substitution was justified by the observation that the SRC is located at the surface of HDAC3 and exhibits a diverse range of structural skeletons; thus, modifications in this region do not significantly affect HDAC3 inhibition. Likewise, regarding PD-L1 interaction, the essential structural motif was identified as the biphenyl pharmacophore of Wang-24,^82^ while the tail group extends outside the PD-L1 dimer, making it suitable for the insertion of various alkyl chain types.

Based on this rationale, the present research group developed a series of dual compounds in which the pyrido-biphenyl core of Wang-24 was fused with an *N*-propylated hydrazide by interposing different types of linkers (aromatic or aliphatic), among which compound **31** (**PH3**) showed the most promising biochemical inhibition profile (Figure 10). **31** displayed 94.3% inhibition of PD-1/PD-L1 interaction at 1 μM, along with an IC_50_ value of 0.107 μM against HDAC3. Its selectivity over HDAC1 (>20-fold) and HDAC6/8 (>90-fold) was also confirmed. Interestingly, although the HDAC-targeting portion of **31** lacks the typical alkylated benzoyl hydrazide pharmacophore, it still exhibits preferential inhibition toward HDAC3. The antiproliferative activity of **31** was evaluated on B16–F10 (murine melanoma cell line), A375 (human melanoma cell line), HepG2 (hepatocellular carcinoma), 4T1 (mouse mammary carcinoma cell line), MDA-MB-231 (human breast cancer cell line), and HCT-116 (human colorectal carcinoma) cell lines, showing significant cytotoxic effects. Notably, the B16–F10 cell type was the most sensitive, as **31** showed an IC_50_ of 2.33 μM. This cell line was then selected for Western blot analysis, where increased total Ac–H3 levels confirmed HDAC3-engagement inhibition. **31** also led to both early and late apoptosis induction, along with cell cycle arrest at the G0/G1 phase.

To confirm the dual HDAC3 and PD-L1 inhibition properties of **31**, its immunoregulatory effects were investigated using the A375/Jurkat T cell coculture model. Administration of **31** enhanced the ability of Jurkat T cells to kill A375 cells compared with the effect on A375 cells alone. Although this result supports the immunomodulatory potential of **31**, a comparison with the administration of single HDAC3 and PD-L1 inhibitors would have been necessary to robustly validate the advantage of the dual compound over combination therapy.^83^

Based on the *in vitro* findings, the *in vivo* antitumor efficacy of **31** was assessed using B16–F10 melanoma mouse models. A dose-dependent inhibition of tumor growth was observed, with stronger effects than the combination therapy of NP19 (PD-L1 inhibitor, see ref. 45) and entinostat. Additionally, tumor-infiltrating lymphocytes (TILs) in treated tissues were analyzed. The infiltration of CD3^+^CD8^+^ and CD3^+^CD4^+^ cells in **31**-treated mice (at 20 mg/kg) was comparable to that observed with NP19 and entinostat administered at 10 + 10 mg/kg (CD3^+^CD8^+^ = 3.34% vs 4.31%, CD3^+^CD4^+^ = 1.89% vs 1.75%). At a higher dose of **31** (40 mg/kg), the level of TIL infiltration increased further, confirming the dose-dependent immunological effect. However, since PD-L1 blockade is expected to activate the entire cancer immunity cycle, a more comprehensive analysis, including other immune cell types such as B lymphocytes and neutrophils, would have been necessary to fully characterize the immunomodulatory profile of **3** **1**.

**Figure 10:**
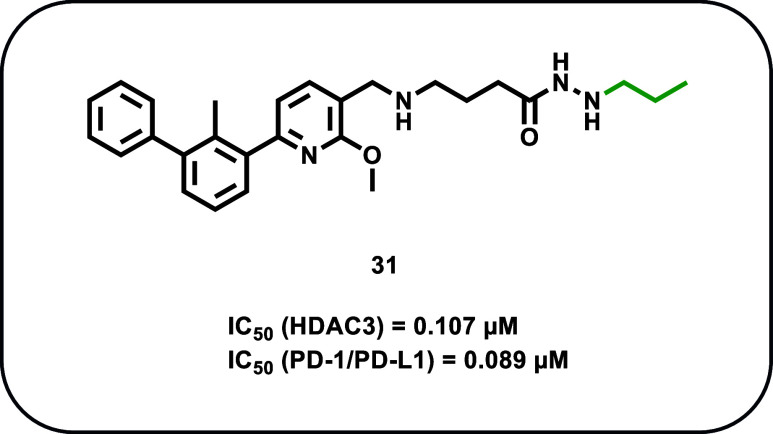
Chemical structure of compound **31**.

## DFMOs: A New Way to Achieve HDAC6-Selective Inhibition

Among the HDAC isoforms, HDAC6 exhibits unique characteristics. While most HDAC6 inhibitors contain HA as their ZBG, none have been yet approved as drugs, primarily due to associated adverse effects.^6^ In addition, Kozikowski and colleagues, through an *in vitro* isotype selectivity screening, have recently observed that HDAC10 is a significant off-target for HA-based HDAC6 inhibitors.^84^

Recently, attention has shifted toward hydroxamic acid-free inhibitors, which might overcome the often-limited isoform selectivity. Among these, the *N*-ethyl hydrazide-based compound **14a** (discussed in paragraph 3.1)^27^ and the difluoromethyl-1,3,4-oxadiazole (DFMO) group have shown promise. The latter, first reported by Kim et al. in a patent in 2017,^85^ demonstrated potent HDAC6 inhibition with IC_50_ values in the low nanomolar range and good selectivity over HDAC1. Even though DFMO appeared in several patents,^86–88^ increased and relevant evidence in the literature have begun in the past few years. Indeed, Onishi et al. first disclosed the DFMO-based selective HDAC6 inhibitor compound **32** (**T-518**) in 2021, which exhibited high HDAC6 selectivity at both the biochemical and cellular levels. In enzymatic assays, it inhibited HDAC6 enzyme activity with IC_50_ values of 0.036 μM (without preincubation) and 0.0046 μM (after 60 min of preincubation). An encouraging pharmacokinetic profile and favorable brain penetration were also observed.^89^ Later, the DFMO derivative compound **33** (**SE-7552**) was reported as an HDAC6-selective inhibitor (IC_50_ = 0.033 μM) with 850-fold selectivity versus all other known HDAC isozymes. Compound **33** demonstrated superior PK compared to hydroxamate-based HDAC inhibitors, and it could block multiple myeloma growth in a xenograft *in vivo* model.^90^ These findings highlight the potential of DFMO-based inhibitors in targeting HDAC6 selectively and effectively.

Initially, the HDAC6 inhibition mechanism played by the DFMO group remained enigmatic, but in the recent years, various research groups^91–93^ have contributed to elucidating its mode of action.

Indeed, it has been demonstrated that DFMO inhibits HDAC6 through a two-step slow-binding mechanism. This involves a Zn^2+^ bound water molecule attacking the sp^2^ carbon adjacent to the difluoromethyl group of DFMOs, leading to the opening of the oxadiazole and the formation of a deprotonated difluoroacetylhydrazide, which acts as the active species implicated in HDAC6 inhibition,^91^ as shown in Figure 11.

**Figure 11:**
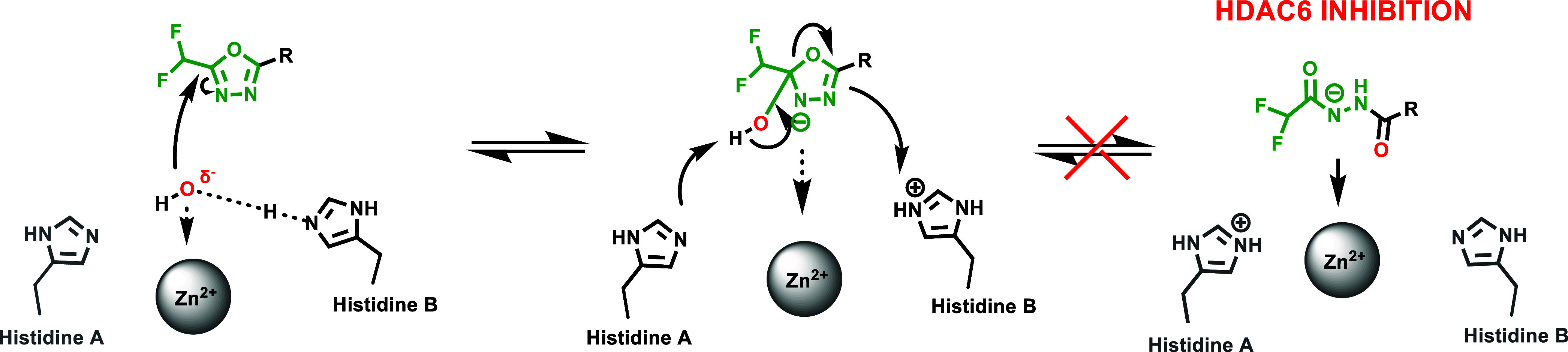
Proposed mechanism of Zn^2+^-catalyzed ring-opening of DFMO to give the corresponding difluoroacetylhydrazide.^91^

The evidence that DFMOs are HDAC6 mechanism-based inhibitors was first derived from the analytical studies conducted by the Steinkühler research group (Italfarmaco SpA), which combined kinetics, X-ray crystallography, and mass spectrometry assays to confirm the DFMO role as an HDAC6 slow-binding substrate after undergoing two hydrolytic steps catalyzed by HDAC6.^92^ Indeed, they confirmed this process by analyzing the mechanism of DFMO’s activation and the consequent HDAC6’s inhibition of a DFMO-based HDAC6 inhibitor compound **34a (ITF5924)** developed by the Caprini research group.^94^ The former exhibited an HDAC6-inhibiting potency in the single-digit nanomolar range (IC_50_ = 0.008 μM) while sparing the HDAC10 inhibition (IC_50_ > 100 μM). Indeed, the refined electron density of the crystallized structure of **34a** within HDAC6 did not match the inhibitor structure, thus suggesting that the DFMO ring-opening reaction occurred. By trying to fit different kinds of substructures derived from the DFMO ring-opening with the obtained electron density, they saw that the best fit was achieved when a hydrazide moiety, instead of the DFMO group, was inserted into the molecular structure of **34a**. So, the initial DFMO’s hydrolysis afforded the corresponding difluoroacetylhydrazide, which acted as the HDAC6 inhibitor, but, subsequently, a secondary hydrolysis occurred, generating the corresponding primary hydrazide which created a tight and long-lived HDAC6-inhibitor complex. Interestingly, to give further insight into the inhibiting interaction within HDAC6’s active pocket, they reported a crystallographic structure of the complex between HDAC6 and the hydrazide inhibitor **34b**, resulting from HDAC6-mediated hydrolysis of the same compound **34a**, in which it was possible to appreciate the main interactions within the catalytic site (Figure 12).

**Figure 12:**
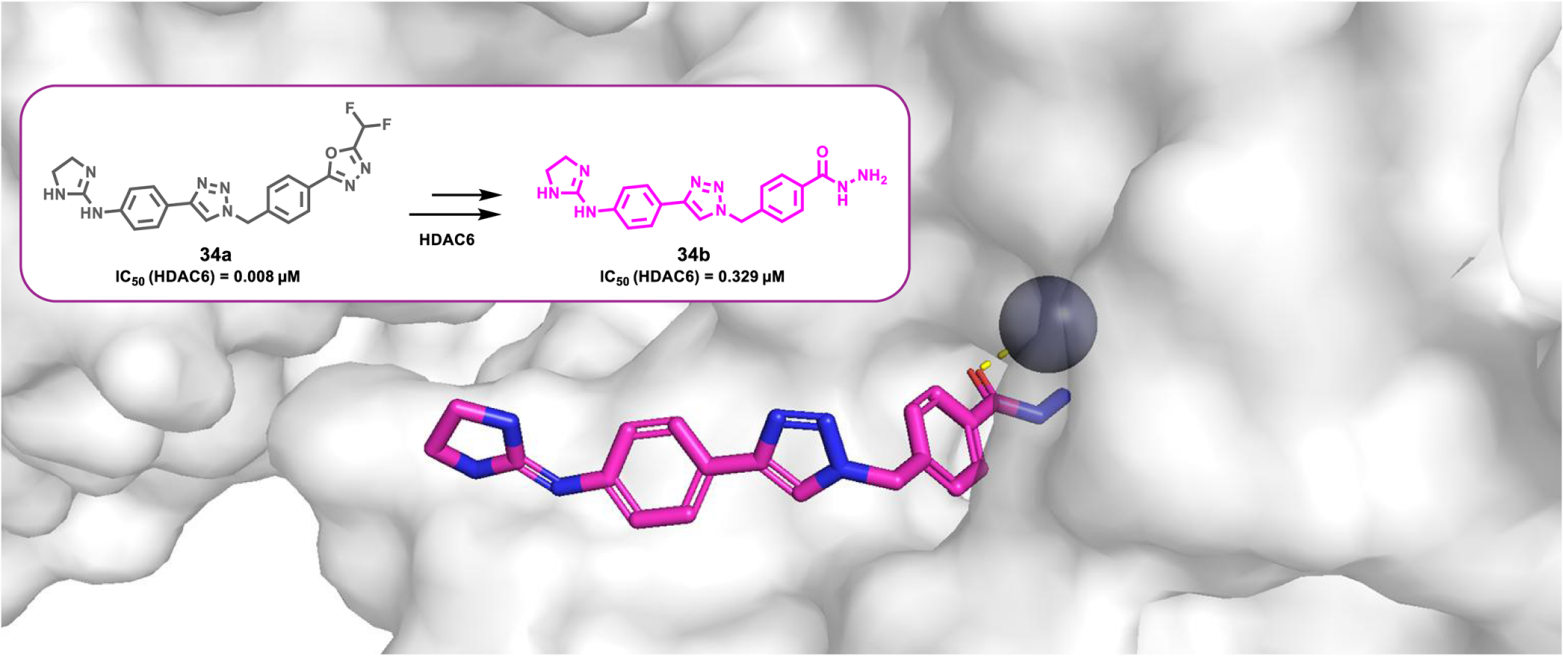
Crystal structure of compound **34b** (magenta) in complex with HDAC6. Polar contact within zinc cation (gray) is displayed (PDB: 8A8Z).^92^

In a parallel study, the Barinka research group performed advanced analyses employing quantum mechanics/molecular mechanics (QM/MM) to confirm the double hydrolysis of the DFMO oxadiazole catalyzed by HDAC6.^95^ Indeed, they provided additional evidence that DFMOs can undergo an enzyme-catalyzed ring-opening reaction to a difluoroacetylhydrazide, as well as a significantly slower second hydrolytic step to the corresponding unsubstituted hydrazide. To better understand the influence of the difluoromethyl group in the oxadiazole ring of DFMOs in modulating HDAC6 inhibition, they first synthesized a series of non-, mono-, di-, and trifluorinated functionalized 1,3,4-oxadiazoles and the corresponding hydrazide-based derivatives (Figure 13). All the synthesized compounds were tested against all HDAC isoforms, and how predictable from the above-discussed DFMOs SAR, compounds harboring the difluoromethyl and trifluoromethyl- 1,3,4-oxadiazole exhibited the best HDAC6-inhibiting and selective profile Compound **35a**, which bears a difluoromethyl group, was the most potent inhibitor in their developed series, showing the highest HDAC6 inhibitory potency (IC_50_ = 0.003 μM) and 1000-fold selectivity for HDAC6 over the other tested HDACs (HDAC1–11). Interestingly, compound **35b**, which contained a trifluoromethyl group, exhibited potent HDAC6-inhibiting properties, as well (IC_50_ = 0.015 μM), with more than 150-fold selectivity over the other isoforms. The monofluorinated derivative, compound **35c**, displayed lower potency against HDAC6 inhibition (IC_50_ = 0.085 μM), whereas the methyl-substituted analog, compound **35d**, showed no activity against HDAC6, even at a 100 μM concentration. The corresponding hydrazide **35e** highlighted a very weak inhibition against HDAC6, thus confirming the trifluoromethyl-1,3,4-oxadiazole and the DFMO rings as preferred moieties for achieving potent inhibition against HDAC6.^95^

**Figure 13:**
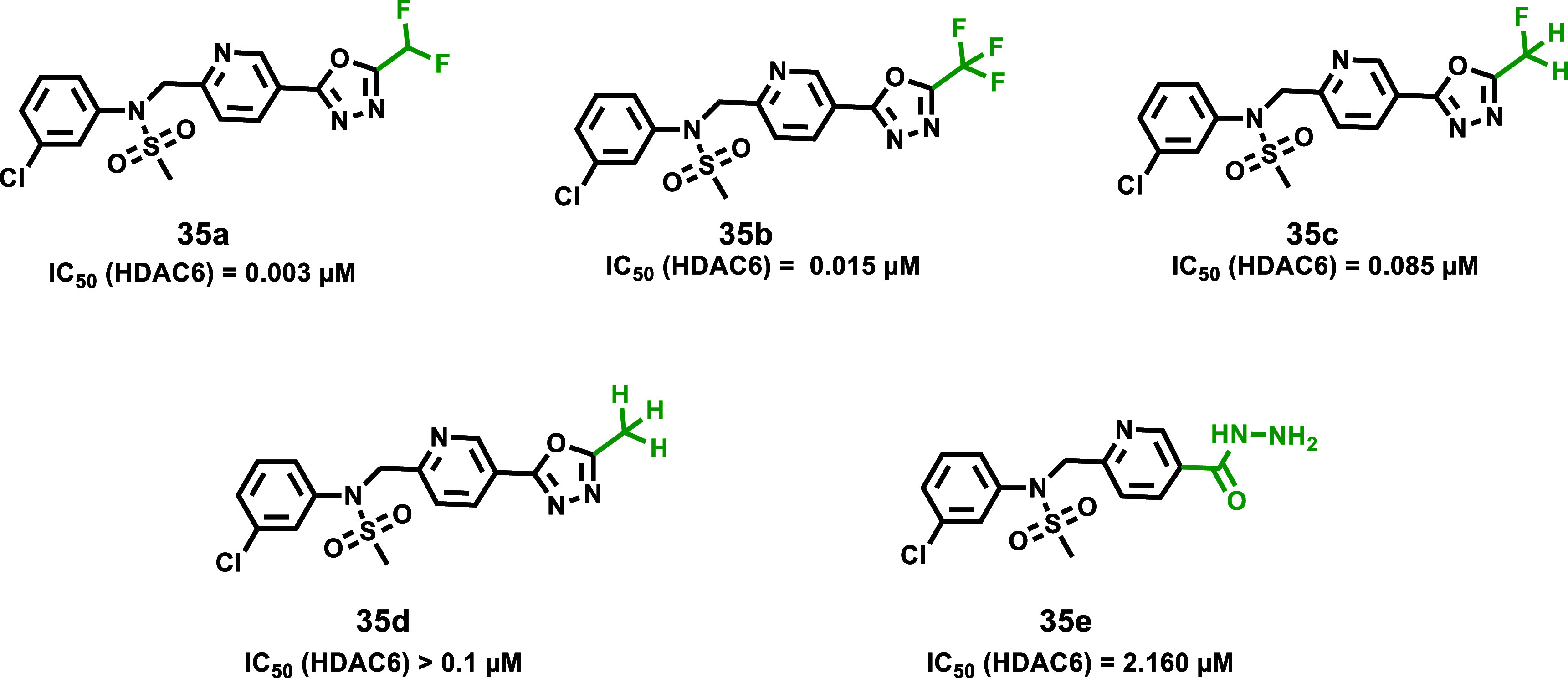
Chemical structures of DFMO-containing compounds **35a**–**e**.

To complete their study, the binding mode of the best DMFO-prototype **35a** to HDAC6 was analyzed by exploring the interaction between the catalytic Zn^2+^ of HDAC6 and the corresponding open hydrazide **35e**, derived from **35a**.^95^ The cocrystal structure of compound **35e** and CD2 (*z*HDAC6) complex revealed bidentate coordination between the hydrazide group (C=O and the distal-NH_2_) and Zn^2+^ (Figure 14).

**Figure 14:**
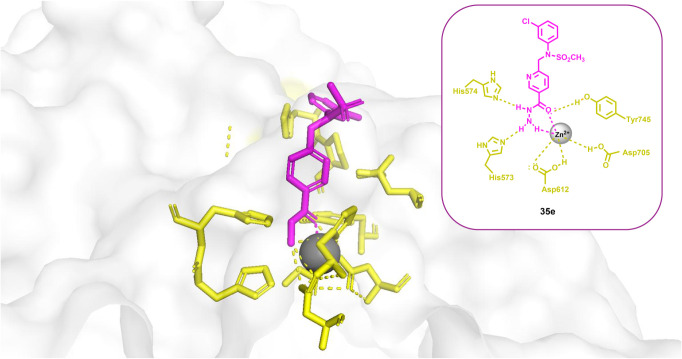
Crystal structure of compound **35e** (magenta) in complex with HDAC6. Polar contacts within zinc cation (gray) and the active site’s residues (yellow) are displayed (PDB: 8BJK).^95^

In the same year, the Christianson and Hansen groups thoroughly investigated the properties of DFMO-based HDAC6i by disclosing the full experimental details of compound **36a** (**BK1**)^96^ confirming that the DFMO warhead undergoes an enzymatic ring-opening reaction, resulting in a deprotonated difluoroacetylhydrazide (compound **36e**) as the active species,^91^ which strongly interacts through an anionic coordination with the HDAC6’s Zn^2+^ in its P571 pocket, resulting in an essentially irreversible enzyme inhibition (Figure 15). HDAC6 inhibition assays with **36a** showed a submicromolar IC_50_ value (IC_50_ = 0.193 μM), with a <15% inhibition at 10 μM against HDAC1–4. However, inhibition activity against HDAC10 should be investigated to fully ascertain the HDAC6 selectivity of compound **36a**.

**Figure 15:**
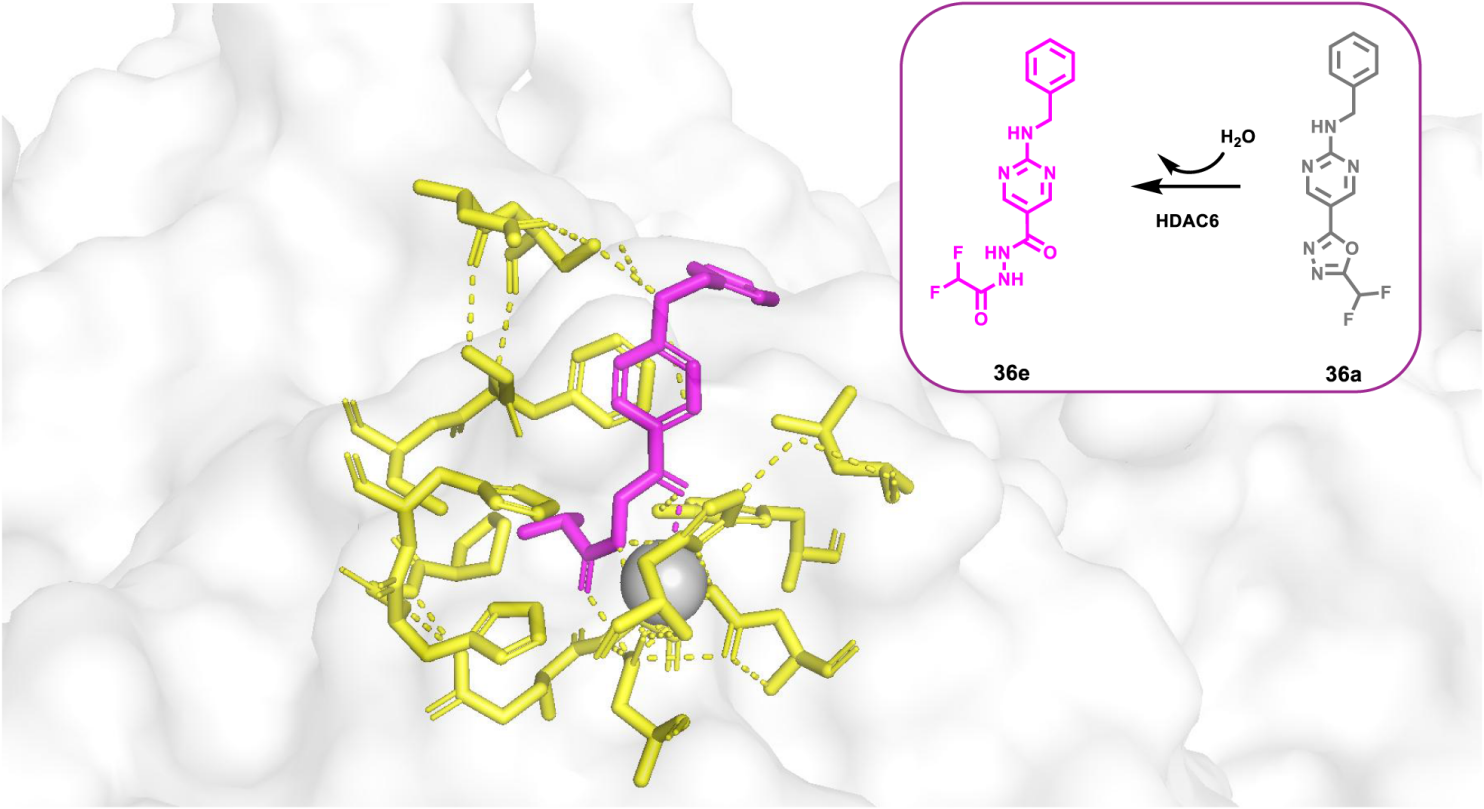
Crystal structure of compound **36e** (magenta) in complex with HDAC6. Polar contacts within zinc cation (gray) and the active site’s residues (yellow) are displayed (PDB: 8GD4).^91^

In line with the Barinka research group’s work,^95^ to better understand the SAR properties given by DFMO in HDAC6-selective inhibitors, the Christianson research group synthesized derivatives of compound **36a**, containing, respectively, a trifluoromethyl-1,3,4-oxadiazole (**36b**), a monofluoromethyl-1,3,4-oxadiazole (**36c**), a methyl-1,3,4-oxadiazole (**36d**) and a difluoromethylacylhydrazide (**36e**) (Figure 16). Biochemical assays showed that compounds **36d** and **36e** had very low HDAC6-inhibiting activities (<15% inhibition at 10 μM). Compound **36c** also displayed weak HDAC6-inhibiting properties (54% inhibition at 10 μM), while compound **36b** exhibited a good HDAC6-inhibiting potency (IC_50_ = 0.531 μM) by showing also weak inhibition properties toward HDAC1–4 (<15% inhibition at 10 μM). Considering the obtained results, the authors suggested that the C=N bond of the oxadiazole ring within compounds **36c** and **36d** may not be sufficiently reactive for nucleophilic attack by the HDAC6’s Zn^2+^-bound water molecule. Furthermore, assays conducted with different preincubation times showed that **36a** and **36b** followed different inhibition mechanisms: the former acted via a two-step slow-binding mode, while the latter followed a single-step slow-binding mechanism.^91^

**Figure 16:**
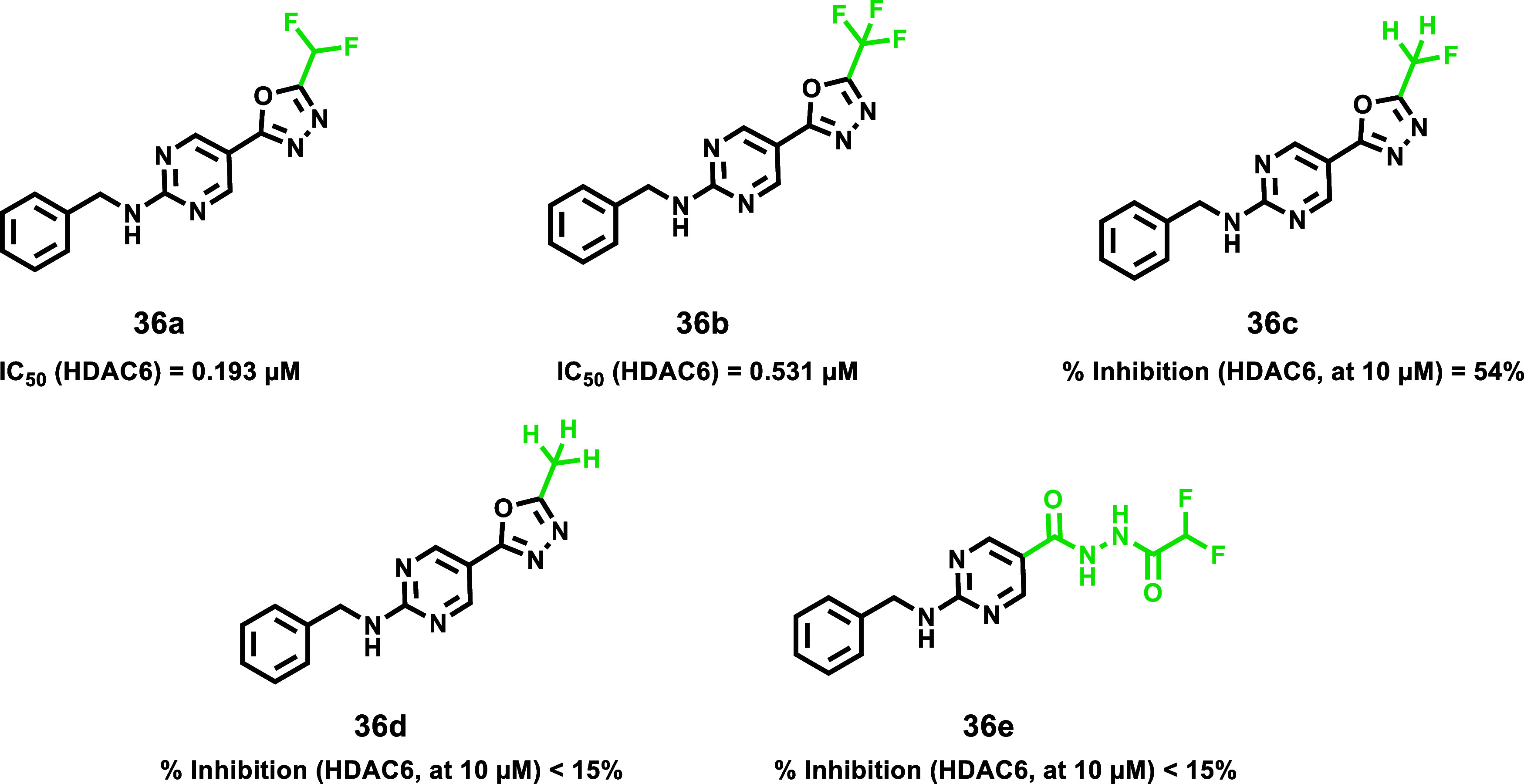
Chemical structures of DFMO-containing compounds **36a**–**e**.

Recently, Ripa and colleagues further corroborated the above findings by synthesizing a series of DFMO-based HDACi that underwent enzyme biochemical evaluation. Indeed, from a HTS campaign against human full-length recombinant HDAC6 of 1.8 million compounds taken by the AstraZeneca chemical library, compound **37** (Figure 18), bearing a DFMO group, was identified as a potent HDAC6 inhibitor (IC_50_ = 0.002 μM). However, the former exhibited poor physicochemical properties, showing low aqueous solubility in phosphate buffer at pH 7.4 and high turnover rate in human microsomes. To improve compound **37**’s properties, Ripa et al. developed a series of 2-(difluoromethyl)-1,3,4-oxadiazole-based HDAC which displayed high oral bioavailability and low *in vivo* clearance.^93^ Among their developed series, compound **38** displayed high potency against HDAC6 (IC_50_ = 0.001 μM), selectivity over HDAC10 (IC_50_ > 100 μM), good ligand-lipophilicity efficiency (LLE), and higher water solubility properties than its parent compound **37**. To further confirm the HDAC6 selectivity over other HDAC isoforms, compound **38** was biochemically tested against a panel of HDACs, and no inhibition up to a 100 μM concentration was detected for any HDAC isoform except for HDAC6. According to the previously discussed reports, the authors confirmed that a prolonged incubation time with the DFMO-containing compounds led to an increased HDAC6-inhibiting potency. Indeed, compound **38** proved an IC_50_ = 0.027 μM after 30 min of incubation time but displayed a 25-fold improvement of the inhibition potency (IC_50_ = 0.001 μM) when the incubation time was extended to 20 h.

Recently, a deeper understanding regarding the second hydrolysis, which converts the acyl-hydrazide to the corresponding hydrazide, has been obtained by Cellupica et al. thanks to additional NMR and LC-MS kinetic studies regarding the hydrolysis process of a novel DFMO inhibitor, compound **39** (**ITF7209**) and the previously discussed compound **34a**.^97^ Compound **39** is an HDAC6 inhibitor (IC_50_ = 0.007 μM) that exhibited selectivity over HDAC1 (IC_50_ > 100 μM), however lacking data regarding other HDACs, such as HDAC10. As previously stated, the first DFMO’s hydrolysis leads to the formation of an acyl-hydrazide, which remains bound to the HDAC6 active site via a strong coordinate bond (N → Zn^2+^) and other noncovalent interactions (NCIs). In the end, such interactions result in essentially irreversible inhibition of HDAC6.

The second hydrolysis may proceed through one of two mechanisms (Figure 17):

1. **Dissociation of the acyl-hydrazide (a)** allowing a second catalytic water molecule to restore the Zn^2+^ coordination sphere.
2. **Direct** ***in situ*** **hydrolysis of the acyl-hydrazide (b)**, where the molecule remains bound to the active site. This possibility is supported by computational analyses performed by Barinka and colleagues.^95^

**Figure 17:**
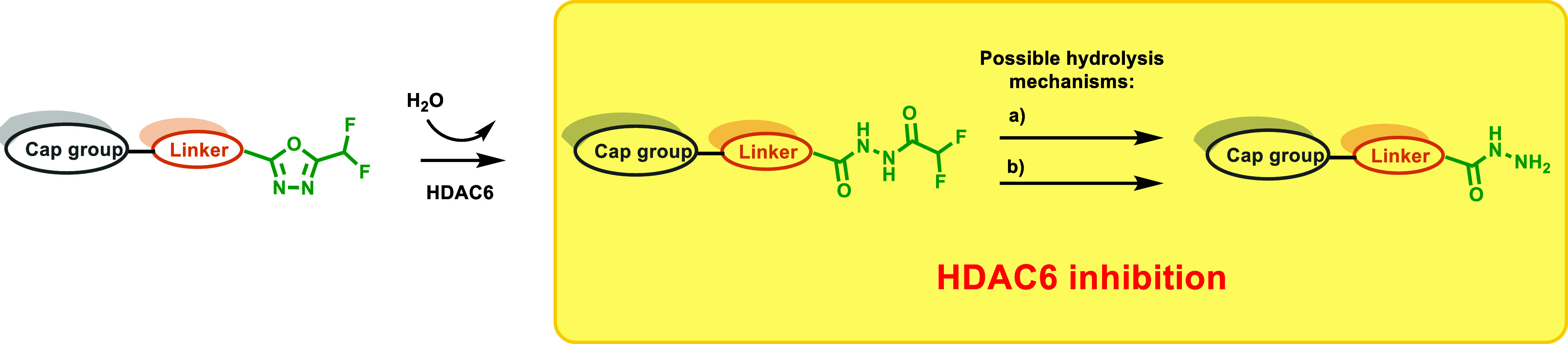
General mechanism of HDAC6 inhibition by DFMOs.

Although the first mechanism appears to be predominant, as evidenced by NMR studies, the coexistence of both mechanisms during the second hydrolysis step has been observed. Enzyme kinetic studies conducted via NMR with compounds **34a** and **39** unveiled distinct behaviors. Compound **34a** underwent the second hydrolysis step through both proposed mechanisms, whereas compound **39** followed only the first mechanism. This difference lies in the structural complexity of the compounds (Figure 18). Compound **34a**, being structurally more intricate, establishes stronger interactions within the HDAC6 active site, enabling both pathways. Conversely, compound **39**, with its simpler structure, engages in fewer interactions, restricting hydrolysis to the first mechanism. Therefore, the pathway by which HDAC6 proceeds to release the corresponding hydrazide from the DFMO-based compound depends primarily on the compound’s structure and its mode of interaction with the enzyme.^97^

**Figure 18:**
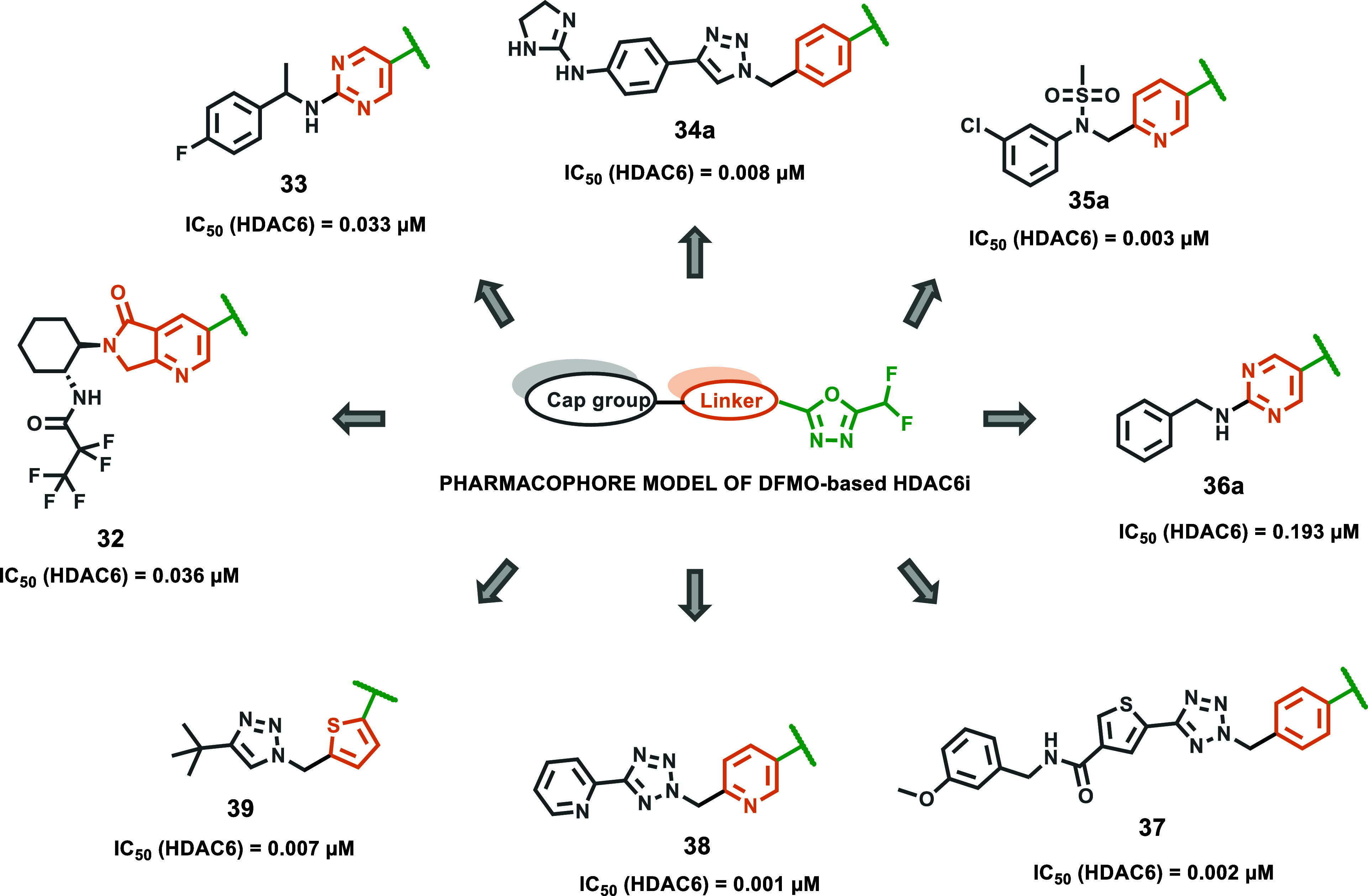
Chemical Structures of DFMO-based HDAC6 inhibitors.

As already discussed, Ripa et al. also focused on DFMOs’ pharmacokinetic properties, confirming a high oral bioavailability, low *in vivo* clearance properties, and neither cardiovascular toxicity nor genotoxicity. However, they observed that DFMOs were stable at pH = 7 but chemically unstable at acidic and basic pH values. Indeed, due to DFMOs’ electrophilic properties, they are susceptible to degradation in water solutions, potentially generating toxic products, thus potentially limiting their applicability especially for chronic diseases.^93^ For this reason, even if DFMO usage in the development of HDAC6 inhibitors to target cancer may represent a successful therapeutic treatment, DFMO-based HDACi need further structural optimization to let them be applicable in chronic disease treatments, in which high adverse effects could not be tolerated.

## Conclusions

HDACs are critical therapeutic targets in cancer and various chronic diseases, and their inhibition could have significant and important therapeutic benefits. So far, several HDAC inhibitors have undergone clinical trials, with five receiving FDA approval for cancer treatments (vorinostat, panobinostat, romidepsin, and belinostat) and for the treatment of Duchenne muscular dystrophy (givinostat). Over the past decades, the development of HDACi has primarily focused on well-established pharmacophore models, using mainly HA and 2-aminoanilide as ZBGs. However, despite the efficacy achieved, many of these compounds have shown metabolic liabilities contributing to toxicity in *in vivo* models.

In response to these challenges, novel ZBGs have been explored, with hydrazides emerging as a promising and safer alternative since their first report in 2015.^23^ Detailed SAR studies have elucidated key features of hydrazide-based HDACi. Notably, optimal inhibitory potency has been achieved with monoalkylated hydrazides bearing at the distal nitrogen linear aliphatic chains of differently sized lengths. Such substitutions confer selectivity for specific HDAC isoforms by exploiting the additional foot pockets present in the active sites of certain HDACs. For example, *N*-ethyl, *N*-hexyl, and *N*-palmitoyl substitutions favor selectivity for HDAC6, HDAC8, and HDAC11, respectively, while *N*-propyl and *N*-butyl chains led to HDAC1–3 selectivity. However, the incorporation of structural moieties such as hydrazides must be done with a detailed understanding of the impact on both the pharmacokinetic and pharmacodynamic profiles.

Indeed, to obtain selectivity among the HDAC1–3 isoforms, further refinements of the *N*-propyl-or *N*-butyl-based HDACi by modifying their cap group led to enhanced enzyme-specific interactions, a strategy particularly effective for the design of HDAC3-selective inhibitors. The promising properties of hydrazides have also been investigated during the development of HDAC-based dual inhibitors, this being a field of great interest in medicinal chemistry, preclinical, and clinical settings, considering the current clinical trials ongoing with the dual HDAC/TKR hybrid inhibitors CUDC-101 (NCT01171924, NCT01702285, NCT00728793, and NCT01384799) and fimepinostat (CUDC-907; NCT02307240, NCT02909777, NCT03002623, NCT02674750, NCT01742988, and NCT03893487). Fimepinostat gained in 2018 Fast Track designation by the FDA in adult patients with relapsed or refractory diffuse large B-cell lymphoma (DLBCL) after two or more lines of systemic therapy, however it has not yet been finally approved.^98^ Indeed, hydrazides might offer significant advantages in this context, as their physicochemical properties and flexibility facilitate the incorporation of functional groups to address additional therapeutic targets without compromising the HDAC-binding efficiency. However, the present hydrazide-based dual-targeting compounds are very recent and early examples that need further validation *in vitro* and *in vivo* for efficacy and safety.

Consequently, hydrazides present a considerable advancement in HDAC inhibitor development, enabling the design of molecules tailored to specific isoforms and dual therapeutic applications. Their favorable pharmacokinetic profiles and tunable selectivity hold promise for targeting isoforms linked to cancer, chronic inflammatory disorders, and immunomodulation, such as HDAC3’s role in PD-L1 expression. Additionally, their utility has also been proven in developing PROTAC-based derivatives, in which selective HDAC3 or HDAC8 degradation has been achieved.^67–70^

Although alkyl hydrazides have recently emerged as promising ZBGs in the development of HDAC inhibitors, offering a potentially safer and more selective alternative to classical hydroxamic acids, their metabolic fate remains only partially understood. To date, most of the available data on hydrazide metabolism and related toxicity come from well-characterized drugs such as isoniazid and iproniazid, both known to induce hepatotoxicity through metabolic pathways that release free hydrazines and aromatic amines, compounds with recognized carcinogenic and toxic properties.^99^ Although in-depth studies on the metabolism of hydrazones, such as J147,^100^ have shown no formation of toxic metabolites (e.g., free hydrazines and aromatic amines),^101^ the known toxicity of isoniazid and iproniazid^102^ underscores the need for careful evaluation of the metabolic profile of new HDAC-based hydrazide inhibitors. While currently available preclinical data for this new class of HDAC inhibitors do not report similar metabolic liabilities, further investigations into their biotransformation and potential for toxic metabolite formation are essential to fully characterize their safety profile.

Beyond hydrazides, the incorporation of the DFMO ring has garnered significant attention, particularly for its HDAC6 selectivity. This selectivity stems from the activation of the DFMO group via ring-opening within the HDAC6 active site, thus allowing the DFMO-based HDACi to be selective over HDAC10, a significant off-target for HA-based HDAC6 inhibitors. Although challenges remain, particularly concerning the susceptibility of DFMO-containing inhibitors to acidic or basic aqueous conditions, they represent a critical frontier in HDACi development.

The hydrazides’ increased potency and selectivity translate into a significant reduction in off-target effects, enabling lower therapeutic dosages. This property is particularly beneficial in reducing systemic toxicity and minimizing adverse pharmacological effects often associated with HDACi. As a result, hydrazide-based HDACi could represent an alternative therapeutic option with improved safety profiles, especially for chronic treatments requiring long-term administration. Continued efforts to optimize both the pharmacokinetic and pharmacological profiles of hydrazide- and DFMO-based HDACi are expected to yield novel prototypes with applications in oncology and chronic disease therapies where selectivity and safety are essential.

## Abbreviations

4T1: mouse mammary cell line
A2780: human ovarian carcinoma cell line
A375: human melanoma cell line
AML: acute myeloid leukemia
B16–F10: murine melanoma cell line
BBB: blood–brain barrier
BIM: Bcl-2-like protein 11
Cal27: head and neck carcinoma cell line
CD13: aminopeptidase N
CTSB: cathepsin B
DFMO: difluoromethyl-1,3,4-oxadiazole
DLBCL: diffuse large B-cell lymphoma
FDA: Food and Drug Administration
HA: hydroxamic acid
HCECs: human corneal epithelial cells
HCT-116: human colorectal cell line
HDAC: histone deacetylase
HDACi: histone deacetylase inhibitor
HEK-293: human embryonic kidney-293
HepG2: hepatocellular carcinoma cell line
HL7702: healthy liver cells
HTS: high-throughput screening
LLE: ligand-lipophilicity efficiency
MDA-MB-231: human breast cancer cell line
NCIs: noncovalent interactions
PK: pharmacokinetic
PROTAC: proteolysis targeting chimera
SAR: structure–activity relationship
SHMT2: serine hydroxymethyltransferase
SMC3: structural maintenance of chromosomes 3
SRC: surface recognition cap
TFs: transcription factors
TNBC: triple-negative breast cancer
QM/MM: quantum molecular mechanics
VEGFR: vascular endothelial growth factor
VHL: Von Hippel–Lindau
ZBG: zinc-binding group
